# Quantified pathway mutations associate epithelial-mesenchymal transition and immune escape with poor prognosis and immunotherapy resistance of head and neck squamous cell carcinoma

**DOI:** 10.1186/s12920-024-01818-6

**Published:** 2024-02-08

**Authors:** Yuhong Huang, Han Liu, Bo Liu, Xiaoyan Chen, Danya Li, Junyuan Xue, Nan Li, Lei Zhu, Liu Yang, Jing Xiao, Chao Liu

**Affiliations:** 1 Department of Oral Pathology, Dalian Medical University School of Stomatology, Dalian, China; 2 Academician Laboratory of Immunology and Oral Development & Regeneration, Dalian Medical University, Dalian, China; 3 Institute for Genome Engineered Animal Models of Human Diseases, Dalian Medical University, Dalian, China

**Keywords:** Pathway Mutation Burden (PMB), Tumor Mutation Burden (TMB), Functional genomics, Polyomics, Transcriptome

## Abstract

**Background:**

Pathway mutations have been calculated to predict the poor prognosis and immunotherapy resistance in head and neck squamous cell carcinoma (HNSCC). To uncover the unique markers predicting prognosis and immune therapy response, the accurate quantification of pathway mutations are required to evaluate epithelial-mesenchymal transition (EMT) and immune escape. Yet, there is a lack of score to accurately quantify pathway mutations.

**Material and methods:**

Firstly, we proposed Individualized Weighted Hallmark Gene Set Mutation Burden (IWHMB, https://github.com/YuHongHuang-lab/IWHMB) which integrated pathway structure information and eliminated the interference of global Tumor Mutation Burden to accurately quantify pathway mutations. Subsequently, to further elucidate the association of IWHMB with EMT and immune escape, support vector machine regression model was used to identify IWHMB-related transcriptomic features (IRG), while Adversarially Regularized Graph Autoencoder (ARVGA) was used to further resolve IRG network features. Finally, Random walk with restart algorithm was used to identify biomarkers for predicting ICI response.

**Results:**

We quantified the HNSCC pathway mutation signatures and identified pathway mutation subtypes using IWHMB. The IWHMB-related transcriptomic features (IRG) identified by support vector machine regression were divided into 5 communities by ARVGA, among which the Community 1 enriching malignant mesenchymal components promoted EMT dynamically and regulated immune patterns associated with ICI responses. Bridge Hub Gene (BHG) identified by random walk with restart was key to IWHMB in EMT and immune escape, thus, more predictive for ICI response than other 70 public signatures.

**Conclusion:**

In summary, the novel pathway mutation scoring-IWHMB suggested that the elevated malignancy mediated by pathway mutations is a major cause of poor prognosis and immunotherapy failure in HNSCC, and is capable of identifying novel biomarkers to predict immunotherapy response.

**Supplementary Information:**

The online version contains supplementary material available at 10.1186/s12920-024-01818-6.

## Introduction

As the seventh malignancy worldwide, Head and Neck Squamous Cell Carcinoma (HNSCC) caused nearly 93,000 incidence and 470,000 deaths in 2020 [1]. Even with the rapid advances in diagnosis and treatment, only 40% of HNSCC patients survived 5 years after incidence [2]. Although immune checkpoint inhibitor (ICI) has shed new light on cancer treatment, through which a variety of cancers have gotten benefits, only 30% of HNSCC patients respond to ICI [3]. As a malignancy with epithelial origin, HNSCC is highly prone to EMT which increases malignancy and immune escape resulting in poor prognosis and ICI resistance. Interestingly, mutations in some driver genes or pathways have been found predictive of HNSCC prognosis and ICI response [4–6]. However, whether these mutations are involved in EMT, and how they enhance malignancy and immune escape remain elusive. The association of mutations in the key pathways or genes with HNSCC EMT and immune escape provides novel perspectives to unveil the biomarkers for HNSCC prognosis and ICI response.

Compared to the changes in copy number and methylation which mainly affect gene expression, somatic mutations are the most widespread genomic alterations affecting not only gene expression, but also gene function. However, in tumorigenesis, the biological context and the heterogeneity of somatic mutations among patients make the functional annotation of somatic mutations extremely complicated. Since somatic mutations are specific in certain pathways or biological processes [7, 8], integrating the somatic mutations in terms of pathways and specific biological processes is an optimal strategy to study the role of somatic mutations in tumorigenesis. Therefore, PMB (also called Pathway Based Tumor Mutational Burden [9, 10], Pathway Instability [11] or Pathway Mutation Perturbation [7], etc.) is proposed and exerts a powerful potential in predicting cancer phenotype (prognosis [7], classification [11], drug response [12, 13] (especially to ICI [7, 10, 14]) etc.). However, current PMB still have limitations, such as the functional redundancy of the selected pathways or gene sets, the concept as population index, as well as the ignorance of the effect of TMB and the location of somatic mutations in pathways.

Although somatic mutations have successfully predicted the responses to targeted drugs, they are less effective than gene expression profiles in predicting cancer phenotype because gene mutations are transmitted through complex networks to influence specific gene expression driving cancer progression [15]. Cancer databases, such as The Cancer Genome Atlas (TCGA), allow scholars to construct and predict associations between genomic mutations and transcriptomic perturbations by providing a wealth of omics data. Way et al. predicted RAS pathway mutations based on the transcriptome using elastic networks [16]; Schubert exploited multi-variable linear regression model to distinguish the responses of specific pathway from tremendous pathway interference [17]; Evan et al. combined multiple network algorithms to propose Tumor Checkpoints [18], the key genes linking upstream mutations to downstream transcriptome perturbations. These examples suggest that Cancer Functional Genomics model of genome-transcriptome-phenotype provides an unique paradigm for cancer system research.

**Fig. 1:**
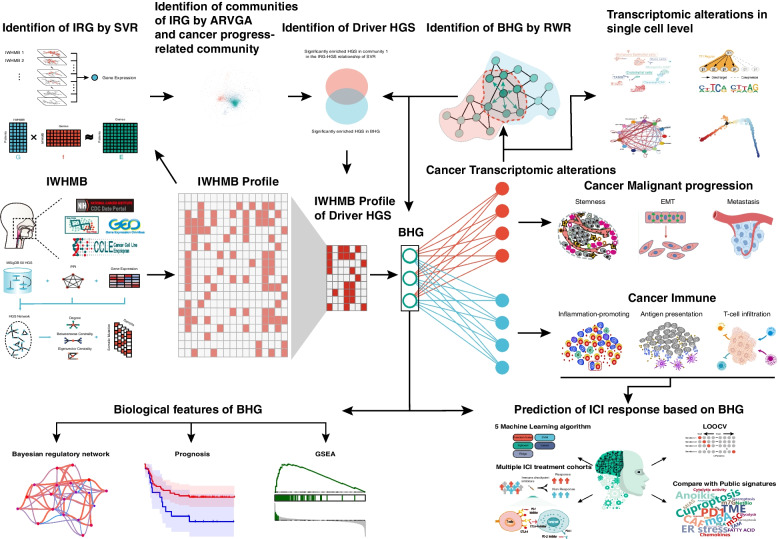
Graphical abstract of the paper design

In this study, we improved an algorithm for PMB calculation and proposed the notion of IWHMB. We developed the R package called IWHMB. Then, by combing machine learning and network algorithms, we revealed the association of IWHMB with cancer phenotype, IWHMB of core pathways and biological processes driving cancer progression and immune transcriptome alterations via BHG. Finally, we prove that BHG outperforms the existing gene signatures in predicting ICI response. The workflow for this study is shown in Fig. 1.

## Materials and methods

### Data source

1 Genomic data (Somatic mutation and copy number variation, Level 3), transcriptomic data (RNA sequencing, Level 3), and the corresponding clinical data of 33 cancers are downloaded from The Cancer Genome Atlas (TCGA) database (https://portal.gdc.cancer.gov/). 2 TCGA HNSCC cohort and Chen’s cohort: data type and download location are same as those of pan cancer cohort. 3 GEO HNSCC cohort: Transcriptomic data and clinical data of five independent HNSCC cohorts were downloaded from Gene Expression Omnibus database (GEO, https://www.ncbi.nlm.nih.gov/geo/), GSE65858, GSE39366, GSE40774, GSE41613, and GSE117973 (Additional file 2: Table S1). 4 Mouse OSCC cell line: Transcriptome data (RNA sequencing, Level 3) are downloaded from GEO database, GSE153383. 5 ICI Cohorts: transcriptome data are obtained from 12 public database ICI cohorts, including IMvigor210 (2018 anti PDL1 Urothelial_Cancer) [19], Braun et al. (2020 PD1_CCRCC) [20], Hugo W et al. (2016 anti PD1_Met_Melanoma GSE78220) [21], Riaz N et al. (2017 anti PD1_Melanoma GSE91061) [22], Aoki H et al. (2021 anti-PD1_STAD GSE154538) [23], Rose TL et al. (2021 ICI_Bladder_Cancer GSE176307) [24]. Liu et al. (2019 anti PD1 Met Melanoma) [25], Prat et al. (2017 anti PD1 NSCLC, HNSCC and Melanoma GSE93157) [26], Gide et al. (2019 anti PD1 + CTLA4 Melanoma) [27], Lauss et al. (2017 ACT Melanoma GSE100797) [28], JaeWon et al. (2020 anti PD1 NSCLC GSE126044), Nathanson et al. (2017 anti CTLA4 Melanoma) [29]. Braun et al. (2020 PD1_CCRCC), Nathanson et al. (2017 anti CTLA4 Melanoma), Liu et al. (2019 anti PD1 Met Melanoma), Hugo W et al. (2016 anti PD1_Met_Melanoma GSE78220) and Lauss et al. (2017 ACT Melanoma) have matching somatic mutation data. 6 Two scRNA-seq data (human HNSCC and mouse OSCC cell line): Transcriptomic data are downloaded from the GEO database, GSE103322 and GSE153383. 7 scTCR seq (mouse OSCC cell line): single cell T-cell receptor sequencing data are downloaded from the GEO database, GSE153383. 8 HNSCC cell gene dependency data (CERES scores for CRISPR knockout screens and METER scores for RNAi screens) are downloaded from Cancer Dependency Map (DepMap, https://depmap.org/) Portal database.

Log (TPM + 1) transformation is performed on bulk transcriptomic data (RNA sequencing) of all cohorts. For transcriptomic data (Gene Expression Array), R package "limma" is used to normalize them. For SNV, only non-silent mutation is retained. For CNV, GISTIC2.0 is used to identify gene level copy number change of recurrent CNVs.

### Calculation of individual weight hallmark gene set mutation burden

Pathway networks were often integrated into global network, which was constantly subject to the interference of different conditions. The concept of individualized treatment suggested that constructing sample-specific network was a better approach, however, the scarcity of such methods and the large variability in network construction across methods make it hard to capture sample-specific networks accurately and consistently. For this reason, cancer-related networks seems a conservative strategy built on the concept of population. Specifically, Protein–Protein interaction (Gene SYMBOL ID) files downloaded from STRING, BIOGRID databases and the parsed KGML file of KEGG database were used to build a global protein–protein interaction network, and reserved the pair with absolute Pearson correlation coefficient > 0.4 and *p* < 0.05 in a cancer cohort to form Cancer Type-Specific protein–protein interaction network.50 HGSs were downloaded from MSiDgb (https://www.gsea-msigdb.org) and mapped to the network built in previous steps. In each HGS network, the three types of node centrality coefficients are defined as:

1 $$Degree=\sum_{i\ne j}{a}_{i,j}$$

2 $$Betweenness\_Centrality=\sum_{s\ne t\ne i}\frac{{P}_{st}\left(i\right)}{{P}_{st}}$$

3 $$Eigenvector\_Centrality=\frac{1}{\lambda }\sum {a}_{i,j}{e}_{j}$$

where Degree, Betweenness Centrality and Eigenvector Centrality are the three Network Centrality Features. For a particular node i in a binary network with n nodes, the Degree represents the number of nodes directly connected to it, Betweenness Centrality estimates the fraction of shortest paths that pass through that node, The Eigenvector estimates the centrality values of the nodes.

Weight HGS Mutation Burden (WHMB) is defined as:

4 $$WHMB=\left\{\begin{array}{c}\frac{1}{{M}_{size}}{\sum }_{\begin{array}{c}gene\in HGS \cap \\ sample mutation genes\end{array}}\begin{array}{c}\left({Degree}_{n}+{Betweennes{s}_{Centraity}}_{n}+{Eigenvecto{r}_{Centrality}}_{n}\right)*gene,\\ gene in network \end{array}\\ \frac{1}{{M}_{size}}{\sum }_{\begin{array}{c}gene\in HGS \cap \\ sample mutation genes\end{array}}gene, gene out of network\end{array}\right.$$

In which

5 $${Centrality\_Feature}_{n}=\frac{Centrality\_Feature-{Centrality\_Feature}_{min}}{{Centrality\_Feature}_{sd}}$$

where M_size_ is number of HGS. Individual Weight HGS Mutation Burden (IWHMB) is defined as:

6 $$\begin{array}{c}IWHMB=\frac{WHMB-{WHMB}_{mean}}{{WHMB}_{sd}}\end{array}$$

$IWHMB$ Zscore $WHMB$ by Sample, $IWHMB$>0 means mutation status of HGS is activated, otherwise it is inactivated or suppressed in a single patient.

EGFR pathways were downloaded from KEGG (https://www.genome.jp/kegg/), GO (https://geneontology.org/) and REACTOME (https://reactome.org/), and combined with 50 HGS into one file to calculate the IWHMB of EGFR pathway.

### Jaccard similarity coefficient

Jaccard similarity coefficient is defined as:

7 $$\begin{array}{c}J\left(A,B\right)=\frac{\left|A\cap B\right|}{\left|A\right|+\left|B\right|-\left|A\cap B\right|}\end{array}$$

### Differential expressed genes (DEGs) analysis

For unpaired samples, analysis of DEGs was performed using the R package DEseq2. For Paired samples, paired t-tests were used, Bonferroni's test was used as a correction for p-value. DEGs confirmation thresholds: high differential genes: abs(log2FoldChange) > 1 & p.adjust < 0.05, medium differential genes: abs(log2FoldChange) > 0.5 & p.adjust < 0.05.

### Enrichment analysis

For DEGs, we performed KEGG and GO enrichment analysis based on the hypergeometric distribution principle (implemented by "EnrichGO" and "EnrichKEGG" in the R package clusterProfiler) with default values. For the List of Gene difference rank (genes arranged in descending order of difference ploidy), we performed Gene Set Enrichment Analysis (GSEA, implemented by "GSEA" in the R package clusterProfiler). For a single sample, we performed Gene Set Variation Analysis (GSVA, implemented by the "gsva" function in the R package GSVA). The gene sets required for GSEA and GSVA analyses were downloaded from the MSigDB database, including three gene sets h.all.v7.5.1.symbols.gmt, c5.all.v7.5.1.symbols.gmt, c2.cp. kegg.v7.5.1.symbols.gmt and the previously reported immune gene set [30]. Enriched pathway Bayesian network inference and visualization is implemented by the R package "CBNplot" [31].

### CIBERSORT immune cell scores

Scores of 22 immune cell are calculated by CIBERSORT through gene exp expression. Tumor Purity and Stromal Score are estimated by ESTIMATE.

### Clustering analysis

Consensus Cluster is implemented by the "ConsensusClusterPlus" function in the R package ConsensusClusterPlus, with parameters set to maxK = 1000, reps = 50, pItem = 0.8, clusterAlg = "km", distance = "euclidean". K-means Cluster is implemented by R base kmeans function. Hierarchical cluster is implemented by R base hclust function. Parameters of K-means Cluster and Hierarchical Cluster are set to default values.

### Tumor mutational burden (TMB) calculation

TMB is defined as the total number of somatic gene coding errors, base substitutions, insertions or deletions detected per million bases, The TMB is calculated using the R package maftools function "maf".

### CNV burden (CNB) calculation

CNB is defined as the sum of absolute value of CNV calculated by GISTIC2 (gene level, amplification: 1 deletion: -1).

### Calculation of stemness based on transcriptomic and methylation data

We took a previously published algorithm [32] to calculate the stemness. The approach is as follows: the predicted model values calculated by previous article are obtained [33] (based on transcriptomic and methylation data of pluripotent stem cell by one-class logistic regression (OCLR) algorithm), then spearman correlation coefficients between our samples and the model predictions are used as the stemness (transcriptome and methylation).

### Community module score

Because the expression changes of each module genes are highly correlated, it makes sense to represent each module by a single representative expression profile called module score. Module score is defined as first principal component of Module Matrix.

### Mutational signatures analysis

The somatic SNVs of each sample were divided into 96-trinucleotide context. Non-negative matrix factorization (NMF) algorithm decomposed it into individual contributions of the reference set of 30 canonical mutational signatures available in the Catalogue of Somatic Mutations in Cancer (COSMIC database; http://cancer.sanger.ac.uk/cosmic/signatures).

### Statistical analysis

For the statistical methods used in this study, Categorical variable correlation analysis: Fisher's exact test with two-sided alternative hypothesis. Two-sided population test; Student's t-test, and Mann–Whitney U-test with two-sided alternative hypothesis. Multiple population test: One-way ANOVA. Rich set analysis (here custom gene sets):Chi-square Test with upper-tailed alternative hypothesis. Relationships between continuous variables were inscribed using Spearman and Peason correlation coefficients. Survival Differences between subgroups were tested using log rank test, and Kaplan–Meier survival curves were generated. The relationship between continuous variables and Overall Survival was tested using Multivariate Cox regression analyses. All statistical analyses were based on R software (4.0.5).

## Results

### Developing IWHMB and exploring its relationship with clinical phenotype in HNSCC

**Fig. 2:**
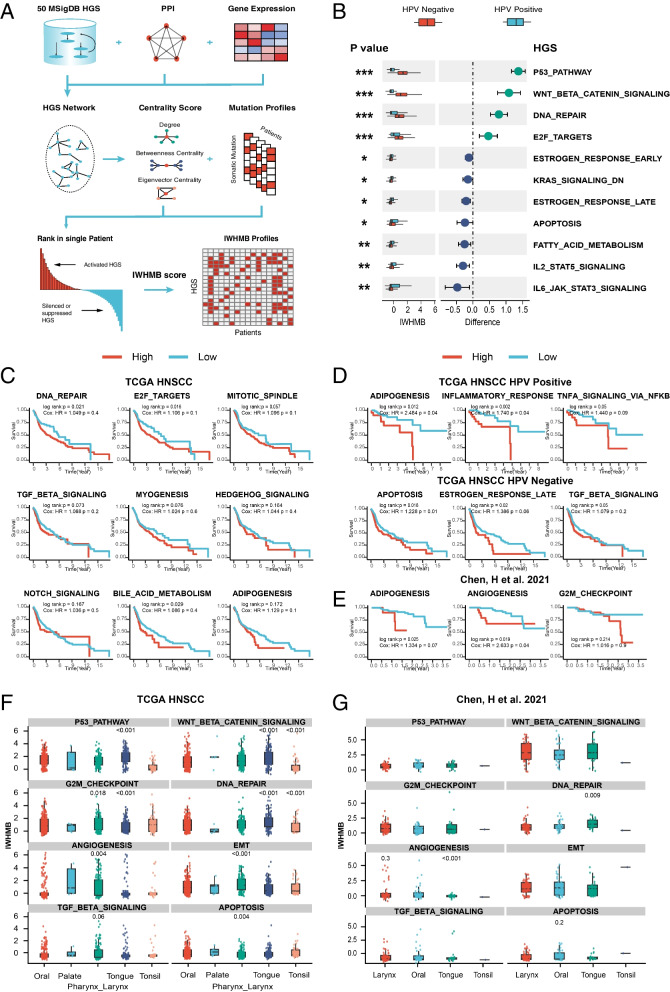
Association of IWHMB with HPV status, clinical prognosis, and subanatomic location in HNSCC **(A)** Pipeline of IWHMB. **B** The magnitude and significant differences in IWHMB according to the HPV status in TCGA cohort. **C-E** IWHMB was significantly associated with prognosis in 4 kinds of HNSCC cohorts (TCGA HNSCC, TCGA HNSCC HPV-negative, positive cohort and Chen’s cohort). **F**, **G** Anatomical location-related IWHMB in two HNSCC cohorts (TCGA HNSCC cohort and Chen’s cohort)

We improved PMB into IWHMB as shown in Fig. 2A. The IWHMB integrated pathway network during counting the number of genomic mutations in the pathway (see Materials and Methods) and ranked at the individual level to eliminate the effect of TMB. This approach outputs an IWHMB matrix (Additional file 2: Table S2 and Additional file 2: Table S3) with rows representing 50 Hallmark Gene Sets (HGS), columns representing patients, and elements representing IWHMB to explore the association of multi-omics with clinical characteristics.

To explore the relationship between IWHMB and the clinical phenotype of HNSCC, we selected HPV status, clinical prognosis and sub-anatomical location, which were to be associated with HNSCC genomic alterations [2, 34]. We found that the IWHMB scores of 11 HGSs were significantly associated with HPV status (t-test *p* < 0.05 in HPV positive and negative subgroups), 4 of which (P53_PATHWAY, WNT_BETA_ CATENIN_SIGNALING, DNA_REPAIR and E2F_ TARGETS) were significantly highly scored in HPV negative subgroup, and 6 of which (IL2_STAT5_SIGNALING, IL6_JAK_STAT3_SIGNALING, FATTY_ACID_METABOLISM, etc.) significantly high in HPV positive subgroup (Fig. 2B). Based on the IWHMB of a single HGS, we divided the patients in two HNSCC cohorts into high (IWHMB > 0) and low score groups (IWHMB <  = 0) to compare the prognostic differences. In TCGA HNSCC cohort, the high score of IWHMB in the cell cycle HGS (DNA_REPAIR, E2F_TARGETS and MITOTIC_SPINDLE), the malignant stroma HGS (MYOGENESIS, HEDGEHOG_SIGNALING, NOTCH_SIGNALING and TGF_BETA_SIGNALING), and sterol metabolism HGS (ADIPOGENESIS, BILE_ACID_ METABOLISM and ESTROGEN_ RESPONSE_LATE) indicated a worse prognosis (log rank:*p* < 0.2, HR > 1) (Fig. 2C). When the TCGA HNSCC cohort was divided into HPV positive and negative groups, the high IWHMB of TNFA_SIGNALING_VIA_NFKB and INFLAMMATORY_RESPONSEHGS implicated a worse prognosis in HPV positive group (log rank:*p* < 0.05, HR > 1), while the high IWHMB of TGF_BETA_SIGNALING and APOPTOSIS suggested a worse prognosis in the HPV negative group (log rank:*p* < 0.05, HR > 1) (Fig. 2D). In Chen’s cohort, the high IWHMB of G2M_CHECKPOINT and ANGIOGENESIS indicated a worse prognosis (log rank:*p* < 0.3, HR > 1) (Fig. 2E). Additionally, sub-anatomical sites also affected IWHMB scores. In TCGA HNSCC cohort, pharyngeal squamous cell carcinoma got the high IWHMB in EMT, ANGIOGENESIS and TGF_BETA_SIGNALING (*p* < 0.05, the mean difference > 0), and a low IWHMB in APOPTOSIS (*p* < 0.05, mean difference > 0) (Fig. 2F). In Chen’s cohort, pharyngeal squamous cell carcinoma got a high IWHMB in ANGIOGENESIS, while oral squamous cell carcinoma got a high IWHMB in APOPTOSIS (*p* < 0.3 and the mean difference > 0) (Fig. 2G). In P53_PATHWAY, WNT_BETA _CATENIN_SIGNALING, G2M_CHECKPOINT and DNA_REPAIR, tongue squamous cell carcinoma had a high IWHMB, while thyroid squamous cell carcinoma displayed a low IWHMB (*p* < 0.05).

Furthermore, we disclosed the tight correlation of IWHMB with clinical stage, metastatic status, tobacco and alcohol consumption in HNSCC patients. The metabolism-related HGS (such as BILE_ACID_ METABOLISM, GLYCOLYSIS and REACTIVE_OXYGEN_SPECIES_PATHWAY) and UV_ RESPONSE_UP got the high IWHMB in early HNSCC (Stage I/II). BILE_ACID_METABOLISM had a consistent tendency in the two cohorts (Additional file 1: Fig. S1A) (TCGA HNSCC: *p* < 0.1, the mean difference > 0; chen’s cohort: *p* < 0.3, the mean difference > 0). In contrast, malignant stroma HGS (such as EMT, ANGIOGENESIS and TGF_BETA_SIGNALING) and cell cycle HGS (such as G2M_CHECKPOINT and E2F_TARGETS) gave the high IWHMB in late HNSCC (Stage III/IV), in which ANGIOGENESIS and G2M_CHECKPOINT showed the consistent changes in the two cohorts (Additional file 1: Fig. S1B) (TCGA HNSCC: *p* < 0.3, the mean difference > 0; Chen’s cohort: *p* < 0.4, the mean difference > 0). Moreover, 3 stemness-related HGS (MTORC1_SIGNALING, HEDGEHOG_ SIGNALING and DNA_REPAIR) displayed the high IWHMB in the metastasis of TCGA HNSCC cohort (Additional file 1: Fig. S1C) (*p* < 0.3, the mean difference > 0). Sterol metabolism HGS (such as ADIPOGENESIS, ESTROGEN_RESPONSE _LATE and ADIPOGENESIS) and APOPTOSIS HGS present the high IWHMB in tobacco or alcohol consumers (Additional file 1: Fig. S1D, E) (TCGA HNSCC: *p* < 0.2, the mean difference > 0; chen’s cohort: *p* < 0.2, the mean difference > 0).

Eventually, we calculated the IWHMB of EGFR pathway to explore the correlation between EGFR signal and HNSCC clinical phenotype based on the IWHMB scoring system (Additional file 1: Fig. S2A). Although the HPV positive HNSCC patients carried less EGFR mutations, the activity of their EGFR pathway were not putatively decreased. Actually, the HPV positive patients in TCGA HNSCC cohorts showed the the higher IWHMB of 3 EGFR pathways than those of HPV negative patients (Additional file 1: Fig. S2B) (*p* < 0.2, the mean difference > 0), which implied that EGFR pathway played differential roles in HPV positive and negative patients. We compared the relationships between EGFR pathway from the 3 gene sets and the prognosis in different HPV status. In the HPV negative group of TCGA HNSCC cohort, the high IWHMB of EGFR pathway from 3 gene sets implicated the worse prognosis, especially the survival period longer than 3 years (log rank:*p* < 0.2, HR > 1). However, in the HPV positive group of TCGA HNSCC cohort, the high IWHMB suggested the better prognosis (log rank:*p* < 0.05, HR > 1). In Chen’s cohort, the gene sets of REACTOME SIGNALING BY EGFR and EPIDERMAL GROWTH FACTOR RECEPTOR SIGNALING PATHWAY showed the high IWHMB correlated with the worse prognosis (log rank:*p* < 0.2, HR > 1), while the high IWHMB of EGFR tyrosine kinase inhibitor resistance was correlated with the better prognosis (Additional file 1: Fig. S2C) (log rank:*p *= 0.18, HR < 1). Meanwhile, we also compared the correlation between the IWHMB of 3 EGFR gene sets and clinical stage and metastasis. In both HNSCC cohorts, the IWHMB of EGFR tyrosine kinase inhibitor resistance was high in late stage (TCGA HNSCC: *p* = 0.01, the mean difference > 0; chen’s cohort: *p* = 0.01, the mean difference > 0). In the TCGA HNSCC cohort, REACTOME SIGNALING BY EGFR and EPIDERMAL GROWTH FACTOR RECEPTOR SIGNALING PATHWAY showed the high IWHMB in metastasis group (Additional file 1: Fig. S2D) (TCGA HNSCC: *p* < 0.3, the mean difference > 0).

### Exploring the association of IWHMB with HNSCC molecular phenotype

**Fig. 3:**
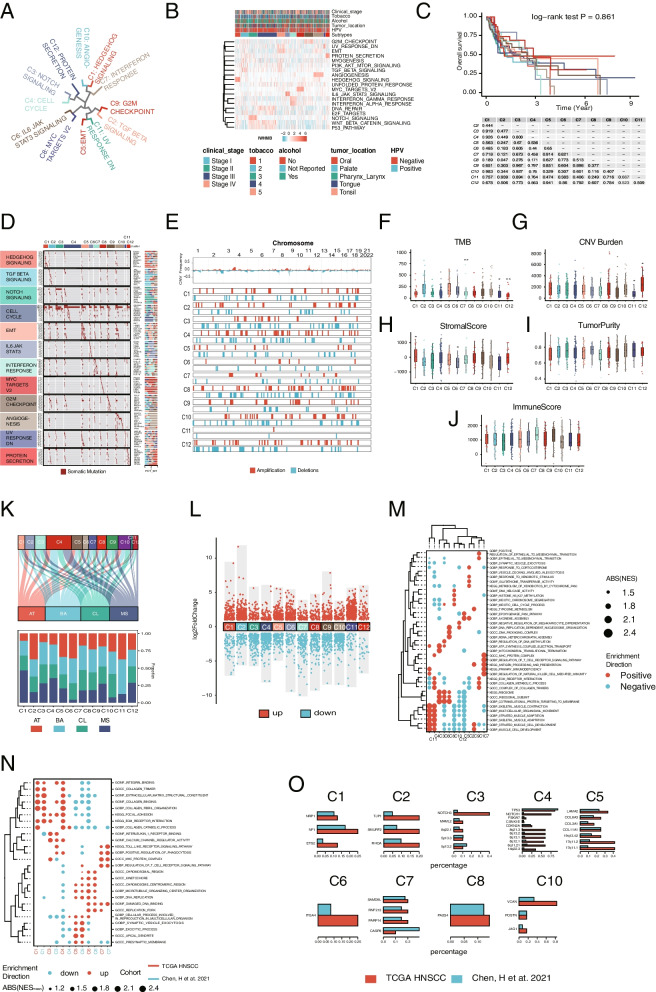
Multi-omics differences in IWHMB-associated cancer clusters in TCGA cohort. **A** Circular cluster dendrogram showing 12 IWHMB-associated cancer clusters. **B** Heatmap showing 12 IWHMB-associated cancer clusters. **C** Clinical prognosis of 12 IWHMB-associated cancer clusters. **D** Somatic mutation waterfall plot of 12 IWHMB-associated cancer clusters. **(E)** Differential copy number changes (Fisher's precision probability test pvalue < 0.05) in 12 IWHMB-associated cancer clusters. **F** TMB of 12 IWHMB-associated cancer clusters. **G** CNV Burden of 12 IWHMB-associated cancer clusters. **H-J** StromalScore, TumorPurity and ImmuneScore of 12 IWHMB-associated cancer clusters. **K** Relationship between IWHMB- associated cancer clusters and Kech clusin of 12 IWHMB-associated cancer clusters. **L** DEGs of 12 IWHMB-associated cancer subtypes. **M** GSEA pathway enrichment of 12 IWHMB-associated cancer subtypes. **N** GSEA-enriched pathways shared by 6 IWHMB-related cancer clusters in two HNSCC cohorts. **O** Shared genomic features of 9 IWHMB-related cancer clusters in two HNSCC cohorts

Hierarchical Clustering was adopted to explore the correlation between IWHMB and molecular features of HNSCC. According to IWHMB clustering, two HNSCC cohorts were divided into 12 clusters (each cluster exhibited one or more IWHMB dominant scores). 9 of the 12 clusters were enriched in the same HGS in two HNSCC cohorts (C1: HEDGEHOG_SIGNALING, C2: TGF_BETA_ SIGNALING, C3: NOTCH_ SIGNALING, C4: CELL CYCLE, C5: EMT, C6: IL6_JAK_STAT3_SIGNALING, C7: INTERFERON RESPONSE, C8: MYC_TARGETS_V2, and C10: ANGIOGENESIS). While the rest 3 clusters were enriched in the different HGS in two HNSCC cohorts (C9: G2M_CHECKPOINT in TCGA cohort and DNA_REPAIR in Chen’s cohort, C11: UV_RESPONSE_DN in TCGA cohort and REACTIVE_OXYGEN_SPECIES _PATHWAY in Chen’s cohort, and C12: PROTEIN_SECRETION in TCGA cohort and UNFOLDED _PROTEIN_RESPONSE in Chen’s cohort) (Fig. 3A, B and Additional file 1: Fig. S3A, B). By comparing the prognosis of 12 clusters, we found that the significant prognostic differences among clusters were only detected in Chen’s cohort (Additional file 1: Fig. S3C), but not in the TCGA HNSCC cohort (Fig. 3C). By extending the comparison to other 32 cancers in TCGA, we found that in 15 cancers (ACC, BLCA, CHOL, KICH, GBM, LGG, LIHC, LUSC, PCPG, PRAD, STAD, THYM, UCEC, UVM and LAML), there were significant prognostic differences among clusters (*p* < 0.05, Consensus Clustering by using the number of clusters with the most significant prognostic differences as k-value, Additional file 1: Fig. S4), implicating that the molecular classification by IWHMB could discriminate the prognostic subtypes of multiple tumors.

Subsequently, we explored the genomic and transcriptomic features of each cluster. As expected, both cohorts showed the significant enrichment of somatic mutations in the representative HGS of each cluster (Fig. 3D and Additional file 1: Fig. S3D). The noticeable heterogeneity of Copy Number Variation (CNV) was disclosed among clusters in both TCGA and Chen’s cohorts. In TCGA cohort, 3q amp region (SOX2, TP63) was mainly enriched in C1, 11q13 (CCND1) in C7, 7p amp region in C4 and C1, 3p del in C12, and 9p del in C8 (Fig. 3E). In Chen’s cohort, 3q amp region was mainly enriched in C4 and C12, 7p amp region in C1, 11q amp region in C4, and 3p del in C4 and C8 (Additional file 1: Fig. S3E). Generally, the CNV heterogeneity was detected not only among the clusters in the same cohort, but also in the same cluster from different cohorts, coinciding to the heterogeneity and diversity among clones during cancer evolution. In TCGA cohort, the TMB of C7, C11 and C12 were significantly lower than that of other clusters (Fig. 3F), while C12 had the highest CNV Burden in all clusters (Fig. 3G), suggesting the CNV dominance during the tumorogenesis of this cluster. In Chen’s cohort, C2 had the lowest TMB in all clusters (Additional file 1: Fig. S3F), while the CNV Burden of C4, C5 and C6 was significantly higher than those of other clusters (Additional file 1: Fig. S3G).

At transcriptomic level, the C1 of both HNSCC cohorts exhibited the highest StromalScore scores based on ESTIMATE algorithm, and the C7 displayed the highest ImmuneScore scores (Fig. 3H-J and Additional file 1: Fig. S3H-J). By comparing the IWHMB clusters with Kech subtypes [35], we found that in both cohorts, C1 had the highest and C6 had the lowest MS enrichment, while C12 had the highest BA enrichment (Fig. 3K and Additional file 1: Fig. S3K). Figure 3L and Additional file 1: Fig. S3L showed the Differentially Expressed Gene (DEGs) (abs(log2FoldChange) > 1 and FDR < 0.05) by comparing the gene expression profile of each cluster with all others. GSEA analysis was performed with the DEGs of each cluster (abs (NES) > 1.5 and FDR < 0.1) (Fig. 3M and Additional file 1: Fig. S3M). The comparison between the GSEAs from the same clusters of two cohorts found that GSEA of C1 was enriched in EMT and Extracellular Matrix (ECM) pathways, C3 in intercellular adhesion and communication pathways, C5 in vesicle secretion and cell membrane component related pathways, C6 in DNA and chromatin structure regulation related pathways, and C7 in immune related pathways (Fig. 3N). The genomic alterations shared by the same clusters in two cohorts suggested that although significantly heterogeneous at genomic level, the IWHMB of clusters exhibited transcriptomic similarity (Fig. 3O), which was detected not only in different clusters within the same cohort, but also in the same cluster from different cohorts. Furthermore, we calculated the contributions of Mutational Signatures in individual sample of TCGA HNSCC cohort with NMF algorithm (Additional file 1: Fig. S5A), and annotated them with COSMIC database (Additional file 1: Fig. S5B). In all clusters, C11 displayed the lowest contributions of Mutation Signatures (liver cancer, DNA mismatch repair, C > T_CpG), C3 and C4 were the lowest in APOBEC mutation signature, while all clusters showed the largest alterations in Tobacco Mutation Signature (Additional file 1: Fig. S5C). Taken together, above findings illustrated that as a progressed PMB, IWHMB was correlated with both clinical and molecular features of HNSCC, and suggested that the IWHMB-related transcriptomic alterations were key to HNSCC phenotype.

### The transcriptomic signatures associated with IWHMB

**Fig. 4:**
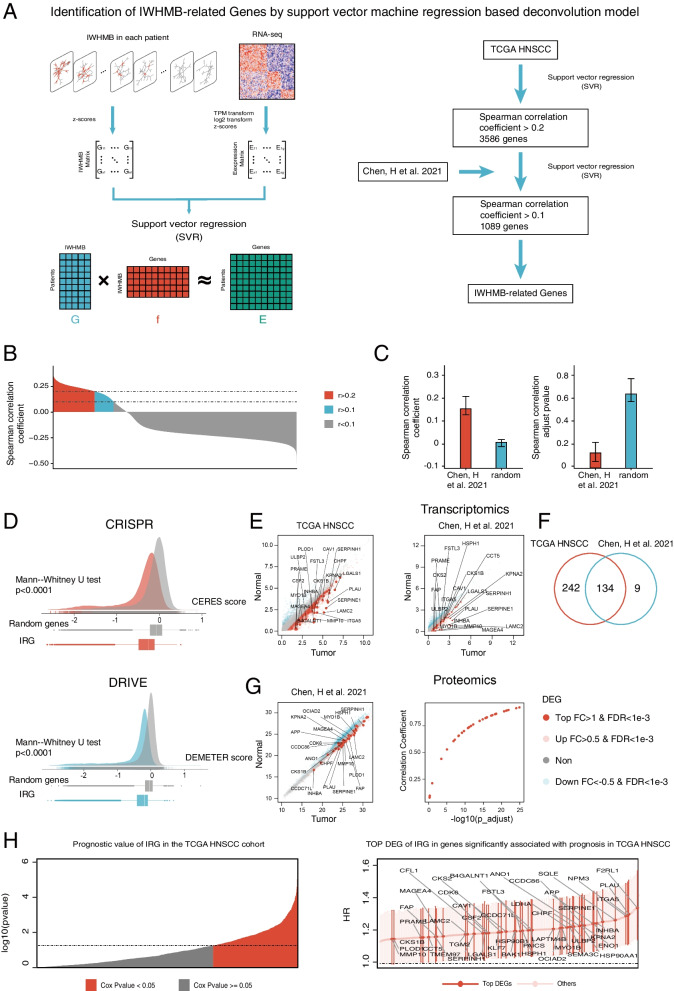
Identification of IRGs.**(A** Deconvolution model based on support vector machine regression identifies IRGs. **B** Speaman correlation coefficients between predictors and their expression levels for all protein-coding genes in the TCGA HNSCC cohort. **C** Further screening obtained IRGs that were conserved in two HNSCC cohorts. **D** The difference of CRISPR- or RNAi-based gene dependency scores between IRGs and random Genes. **E** Differential expressed IRGs in two HNSCC cohorts (tumor vs normal, the most significant IRG is marked). **F** Venn diagram showing the shared differential expressed IRGs genes in two HNSCC cohorts. **G** Differential expressed Proteins in IRGs (tumor vs. normal, the most significant IRG is marked) and their correlation with gene expression in Chen’s cohort. **H** Hazard rations (Cox regression model) of IRGs in the TCGA cohort and their 95% confidence intervals (Top DEGs in all IRGs with significant prognostic relevance were labeled in the proportional hazards model)

To search the IWHMB-related transcriptomic features, we hypothesized that the changes of gene expression were positively correlated with the linear changes of IWHMB. In this hypothesis, because IWHMB scoring of the 50 representative HGSs represented the mutations in upstream pathways, the genes perturbed by the mutations in upstream pathway were regarded as IWHMB-related genes (IRGs), and their expression changes were the transcriptomic signatures associated with IWHMB. To find out the IRGs, we constructed regression models through support vector machine regression (SVR) with a linear kernel function, in which the IWHMBs of 34 HGS were used as the independent variables, and the z-score transformed from the expression of the 21,939 protein-coding genes as the dependent variables (Fig. 4A, See details in Additional file 3: Method 1). We found that the correlation between the most gene expression and the predicted value was too weak to support the direct effects of upstream pathway mutations on most gene expression (Fig. 4B). Therefore, we set correlation coefficients > 0.2 and pvalue < 0.05 as the thresholds and obtained 3586 perturbed genes in TCGA cohort. To find out the conserved genes in both HNSCC cohorts, we further constructed the validating models in Chen’s cohort with the correlation coefficient > 0.1 (pvalue Lower Quartile: 0.034, Upper Quartile: 0.23). Finally, 1089 genes were found conserved in both cohorts (Additional file  2: Table S4). We further excluded the random selection of the 1089 genes in Chen’s cohort by generating a random model in which the mean correlation coefficient between 1089 gene expression and predict value (*r* = 0.0089) was much smaller than that of the validating model (*r* = 0.15), and the mean pvalue (*p* = 0.62) was much larger than that of the validating model (*p* = 0. 13) (Fig. 4C). Then, we disclosed the relationship between IRGs and HNSCC biology. No matter according to CPISPR or RNAi data, IRGs had the significantly lower dependency scores compared to the random gene sets (Fig. 4D). Paired difference analysis screened 376 significantly up-regulated IRGs in TCGA cohort, 143 in Chen’s cohort (Fig. 4E), and 134 in both cohorts (Fig. 4F). Finally, by using the proteomics data of Chen’s cohort for pairwise difference analysis and for the correlation with the transcriptomic data, we found that the protein levels of most significantly up-regulated genes showed a highly positive correlation to the corresponding mRNA levels (Fig. 4G). Cox regression on IRG in TCGA cohort detected a total of 276 DEGs of IRGs with *p* < 0.05, all of which were associated with unfavorable prognosis (HR > 1) (Fig. 4H).

### Determinating communities in IRGs network

In order to disclose how IRGs were associated with HNSCC biology, we integrated the transcriptional regulatory network with the PPI network as described in *Materials and Methods*, and then, mapped IRGs into the integrated network to identify the communities constructed by IRGs. By performing correlation tests for each pair through keeping the pairs with abs(r) > 0.4 and *p* < 0.05, we got a total of 14,387 positive pairs and 249 negative pairs, consisting with the fact that the dominant regulation in cancer was positive feedbacks [36]. Thus, to reduce the noise, only the positive pairs were selected to construct a network with 861 nodes and 14,387 edges. Then, through ARVGA algorithm (Additional file 1: Fig. S6A, see details in Additional file 3: Method 2) in which the loss function value and AUC value of the learning process reached stable (Additional file 1: Fig. S6B), 5 gene collections (hereafter referred to as communities) were detected in the network (Additional file 1: Fig. S6C, Additional file 2: Table S5). We assumed that in the linear model of each gene, the absolute value of regression coefficient > 0.15 represented the gene expression perturbed by the pathway or biological process in HGS (Additional file 2: Table S6). According to this assumption, we plotted the number of genes perturbed by at least two HGSs (Additional file 1: Fig. S6D), and found that most of the IRGs were perturbed by multiple pathways or biological processes in HGSs, the top three of which were P53_PATHWAY (396), G2M_CHECKPOINT (317) and INTERFERON _GAMMA_ RESPONSE (312). These findings further confirmed the heterogeneity in genomic alterations, and the conservativeness in transcriptomic changes. We also found the less overlaps between genes perturbed by HGSs and the genes in HGSs (Additional file 1: Fig. S6E).

### Exploring the association of communities with HNSCC biology and identifying Community 1 as a core transcriptional component affecting HNSCC progression

We further compared the GO and KEGG enrichment among different communities, gene function of Community 1 was mainly enriched in extracellular matrix and hard tissue mineralization pathways, Community 2 in energy metabolism, Community 3 in ribosome-related biological processes, Community 4 in antiviral-related responses, and Community 5 in cranio-maxillo-facial development (Additional file 1: Fig. S7A), suggesting the association of IRGs with multiple HNSCC molecular signatures. Subsequently, according to the CRISPR and RNAi data of HNSCC cell lines in DepMap database, the dependency scores of communities 2 and 3 were the lowest in all communities (Additional file 1: Fig. S7B), indicating the pivotal role of IRGs in HNSCC genesis.. By comparing the coefficient of variation of all 5 communities in multi-HNSCC cohorts (GEO, TCGA and Chen’s cohorts), Community 1, 5, 4, 2 and 3 were lined in the high to low order of the coefficient of variation, in which the lowest coefficient of variation was higher than non-IWHMB-related protein-coding genes (Additional file 1: Fig. S7C). This finding also revealed that the expression of some housekeeping genes, like those in Community 2 and 3, were not fixed, but varied with the progression of HNSCC, endowing them with a potential to be tumor hallmarks [37]. Meanwhile, the comparison of network similarity between communities revealed the topological changes in different HNSCC cohorts, in which Community 1 has the highest network stability, while Community 2 and 3 lowest (Additional file 1: Fig. S7D).

**Fig. 5:**
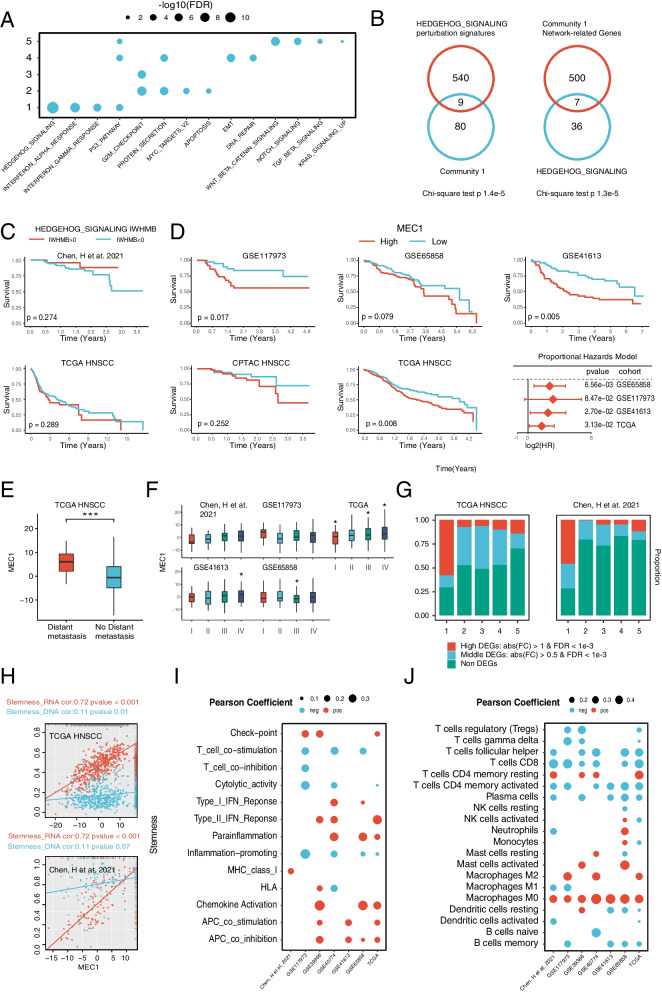
Communities of IRGs **(A)** The association of Communities and HGSs. **B** Right: Relationship between Community 1 and genes disturbed by HH pathway. Left: Relationship between Community 1 related genes confirmed by RWR algorithm and HH pathway genes. **C** Kaplan–Meier curves of IWHMB of HH in high and low groups of TCGA HNSCC and chen’s cohorts. **D** Kaplan–Meier curves and Cox regression models of the MEC1 in multiple HNSCC cohorts. **E** Association of Community 1 with distant metastases in the TCGA HNSCC cohort. **F** Association of Community 1 with clinical stage in multiple HNSCC cohorts. **G** The proportion of differential expressed genes contained in the Communities. **H** Correlation between the MEC1 and two kind of tumor stemness scores. **I** Association of Community 1 with immune scores in multiple HNSCC cohorts. **J** Relationship between Community 1 and the relative content of 22 immune cells in multiple HNSCC cohorts

To find out the community related to HNSCC progression, we focused on Community 1 which had the highest expression variation and network stability. To search the mutations in HGSs driving Community 1, we explored the Community 1 enrichment in different HGSs according to the established correlation between gene expression and HGS in SVR (Fig. 5A). We found that the most significant drive to Community 1 came from Hedgehog (HH) signaling, and the genes in Community 1 were significantly enriched in the gene set interfered by HH signaling. Moreover, in biological network, the first 500 genes adjacent to Community 1 were also significantly enriched in HH signaling (Fig. 5B). All of these results correlated HH signaling mutations to the gene expression of Community 1. Then, we visualized the Community 1 network, and annotated the associated HGSs and KEGG Pathways (Additional file 1: Fig. S7E, F). Intriguingly, although the mutations in HH signaling showed correlation with HNSCC prognosis at some extent, the prognoses predicted by HH signaling mutations showed opposite tendency in TCGA and chen’s cohorts (Fig. 5C). In contrast, Community 1 has a close and consistent correlation with the clinical features of HNSCC. According to the mean values of Module eigenvector of Community 1 (MEC1), we divided the each of TCGA HNSCC, Chen’s and 3 GEO HNSCC cohorts into high and low groups, and found that three cohorts (GSE117973, GSE41613 and TCGA HNSCC) showed the significant survival differences between the high and low groups (log rank *p* < 0.05). Although the other two (GSE65858 and Chen’s cohort) cohorts failed to reach significant differences, the high groups had a remarkable worse prognosis than the low groups. Consistently, Cox regression modeling indicated MEC1 as a cancer risk factor (Hazard Ratio > 1) in 4 of the 5 HNSCC cohorts (Fig. 5D). Furthermore, we found that MEC1 were correlated with distant metastasis and clinical stage of HNSCC. In TCGA cohort, MEC1 were higher in the distant metastases group than those without metastases (Fig. 5E). MEC1 were often higher in the late-stage (Stage VI/III) group in all 3 HNSCC cohorts (Chen’s cohort, GSE41613, TCGA HNSCC) than the early-stage group (Stage I/II) (Fig. 5F).

Further exploration displayed that Community 1 was also associated with HNSCC molecular phenotype. By comparing tumor samples in TCGA HNSCC and Chen’s cohorts with the paired normal samples, the Community 1 displayed the highest proportion in DEGs than other communities (Fig. 5G). Further analysis revealed that the MEC1 exhibited a highly positive correlation with tumor stemness (RNA Stemness in TCGA cohort: *r* = 0.72 and Chen’s cohort: *r* = 0.72; DNA Stemness in TCGA cohort: *r* = 0.11 and Chen’s cohort: *r* = 0.19) (Fig. 5H). Among the ssGSEA scores representing 13 tumor immune processes, the MEC1 had the significantly positive correlation (*r* > 1.5, *p* < 0.05) with 5 immune signatures (Check Point, Type II IFN Reponses, Parainflammation, APC co stimulation and APC co inhibition) and significantly negative correlation with 3 immune signatures (T cell co-stimulation, Cytolytic activity and Inflammation-promoting) in at least 3 HNSCC cohorts (F ig. 5I). Among the 22 immune cells scored by CIBERSORT, MEC1 of Community 1 exhibited the significantly positive correlation (*r* > 2, *p* < 0.05) with 3 immune cells (Macrophages M0, Macrophages M2 and T cells CD4 memory resting) and significantly negative correlation (r < -2, *p* < 0.05) with 4 immune cells (T cells CD4 memory activated, T cells CD8, Plasma cells and T cells follicular helper) in at least 3 HNSCC cohorts (Fig. 5J).

In summary, we found that Community 1, predominantly containing ECM-related genes, was highly expressed in HNSCC compared with the adjacent normal tissues, and highly correlated with various clinicopathological and molecular features of HNSCC. Moreover, the high variability and network stability of Community 1 suggested Community 1 as a core transcriptomic component connecting PMB with the phenotype of HNSCC progression, increasing its potential as a biological marker for HNSCC progression.

### Single cell omics revealed the dynamic promotion of Community 1 to the EMT of HNSCC

To address how Community 1 was closely linked to HNSCC progression, we explored the TME in terms of single cell omics. A total of 5676 HNSCC TME cells from 17 qualified samples (HNSCC tissue and paraneoplastic lymph nodes) of the GSE103322 dataset (Additional file 1: Fig. S8A, See details in Additional file 3: Method 4) were clustered into 8 major cell types, including B cells (PECAM1, SLAMF7 and CD79A), classical-CAF (FAP, PDPN and COL1A2), Endothelial cells (PECAM1 and VWF), TAMM (CD14 and CD163), Malignant Epithelial cells (KRT14 and KRT17), Mast cells (MS4A2 and CMA1), Myogenic-CAF (ACTA2 and ACTG2) and T cells (CD2 and CD3D) (Additional file 1: Fig. S8B, C). The K-means clustering according to the 80 genes in Community 1 divided the mesenchymal cells (annotated as classical-CAF and Myogenic-CAF) and epithelial cells (annotated as Endothelial cells and Malignant Epithelial cells) in HNSCC TME into 8 clusters (ek1-ek8, EMT-related Kmean clusters abbreviated as ek), of which ek1 was characterized by the robust expression of PLAU and LGALS1, ek2 by almost blank expression, ek3 by the robust expression of DDK3, LAMC2 and MFAP2, the expression profiles of ek3 and ek1 were similar but the expression intensity of ek3 was greater, ek4 by the robust expression of P4HA2 and PDPN. ek5 by the robust expression of POSTN, ek6 and ek8 by the robust expression of major genes of Community 1, ek7 by the robust expression of TAGLN (Additional file 1: Fig. S8D).

**Fig. 6:**
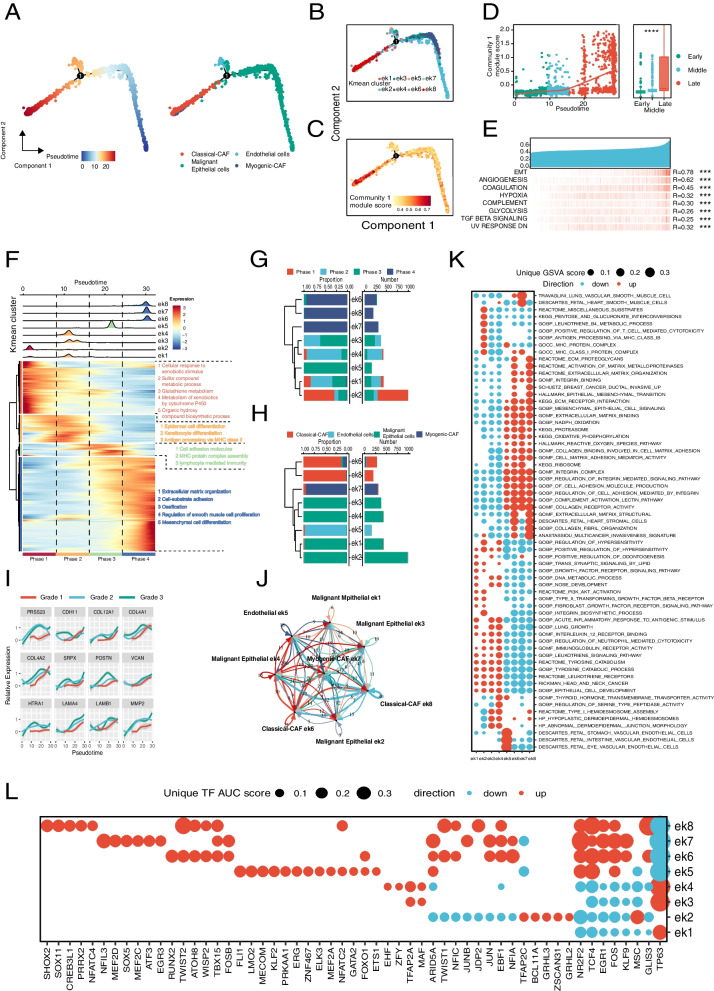
Relationship between Community 1 and EMT in TME of HNSCC **(A)** The trajectory of EMT based on mesenchymal cells and malignant epithelial cells. **B** Distribution of eks along EMT trajectory. **C** Change of Community 1 module score on EMT trajectory. **D** The relationship between Community 1 module score and pseudotime of EMT trajectory. **E** Correlation between the GSVA score of Community 1 and 7 HGSs in single-cell level. **F** Gene heatmap of EMT trajectory and pathway enrichment features in four different periods by pseudotime. **G**, **H** Association of eks with classical cell types and four time periods by pseudotime. **I** Cell-to-cell communication in eks. **J** Evolution of genes in Community 1 associated with pathological grades in EMT trajectories. **K** Biological characterization of eks. **L** Unique transcription factor AUC scores for eks

By constructing pseudo-temporal trajectory with monocle2, we found that Malignant Epithelial cells were distributed in the early stage, Endothelial cells in the middle stage, and CAF in the late stage of the differentiation trajectory (Fig. 6A), implicating the taking place of EMT along trajectory. When ek (Fig. 6B) and Community 1 module score (calculated by Seurat AddModuleScore function) (Fig. 6C) were mapped to the trajectory, it was found that Community 1 module score fit well with the pseudo-temporal progression, in which the score was lowest in the early stage, gradually increasing in the middle stage, and highest in the late stage (Fig. 6D), that associated EMT with the genes of Community 1. By comparing the correlation of GSVA scores between Community 1 and HGSs in TME, the Community 1 GSVA score were highly positively correlated with the GSVA scores of 7 HGSs (EMT, ANGIOGENESIS, HYPOXIA, COAGULATION, GLYCOLYSIS, TGF_BETA_SIGNALING and UV_RESPONSE _DN) (Fig. 6E), which further suggested a comprehensive HNSCC progression enhanced by Community 1 genes.

Further analysis on cellular characteristics divided the pseudo-temporal trajectory into 4 phases. Tanscriptome in phase 1 were dominantly involved in small molecule metabolism (toxic molecules, glutathione, cytochromes, etc.), phase 2 in epithelial differentiation and keratin formation, phase 3 in cell adhesion, MHC complex and immune regulation, and phase 4 in ECM and hard tissue mineralization (Fig. 6F). The ek2 was concentrated in phase 1, ek1, ek3 and ek4 in phase 2, ek5 in phase 3, while ek6, ek7 and ek8 in phase 4 (Fig. 6G). In the term of cell type, ek1-ek4 were constituted by malignant epithelial cells, ek5 by Endothelial cells, ek7 by Myogenic-CAF, ek6 and ek8 by Classical-CAF (Fig. 6H). Consistently, by plotting the trends on the pseudo-timeline, we also found 12 genes in Community 1 associated with HNSCC pathological grading (F ig. 6I). All these findings indicated that an increasing expression of the genes in Community 1 facilitated EMT along trajectory.

Subsequently, CellPhoneDB shows the direct or indirect communications between eks (Fig. 6J). GSVA enrichment analysis revealed that ek1-ek4 showed the high GSVA scores in pathway involved in immune signaling (e.g., interleukins, immunoglobulins, centrocyte toxicity), tyrosine metabolism, epithelial cell development, and HNSCC marker genes, whereas the low GSVA scores in ECM, NADPH, oxidative phosphorylation, ribosome metabolism, protease metabolism, integrins and complement pathways. In contrast, ek5-ek8 exhibited the opposite trends. Furthermore, there are subtype-specific GSVA pathways, such as PIK3-AKT, TGF, and FGF enriched in ek1 and ek2, some immune-related pathways in ek2, and HEMIDESMOSOME pathways in ek3 and ek4, endogenous cellular pathway in ek5, the smooth muscle cellular pathway in ek7, and ECM in ek8 (Fig. 6K). Similar to the GSVA enrichment analysis, SCENIC analysis revealed that compared to ek5-ek8, ek1, ek3 and ek4 were rich in TP63, but short of NR2F2, KLF4 and EGR1; C2 is rich in MSC, TFAP2C and BCL11A, ek3 in TFAP2A and MAF, ek4 in EHF and ZFY, ek5 in FL11 and LMO2, ek6 in RUNX2, TWIST2, etc., ek7 in NFIL3, MEF2D, etc., as well as ek8 in SHOX2 and SOX11 (Fig. 6L). All above results suggested that with HNSCC progression, the single cell transcriptomic features exhibited a robust EMT phenotype mediated by the genes in Community 1.

### Community 1 modified anti-PD1 responses in OSCC immune microenvironment

**Fig. 7:**
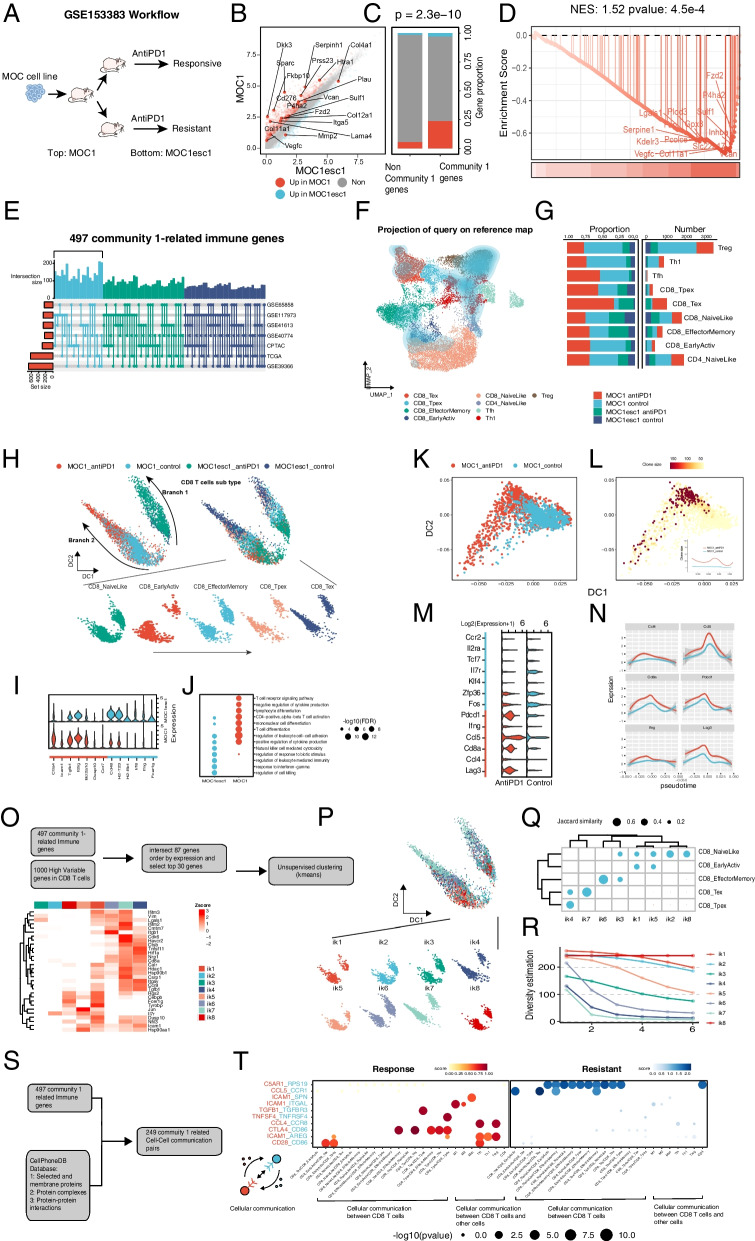
Relationship between Community 1 and features of ICI responses TME of OSCC **(A)** The workflow of the GSE153383 dataset. The MOC cells from mouse OSCC line were injected into mice which were subsequently subjected to anti-PD1 therapy. The MOC line produced in responding mice was MOC1, and the MOC line in resistant mice was MOC1esc1. **B** DEG analysis of MOC1 and MOC1esc1. **C** Distribution of DEG in Community 1 and non-Community 1. **D** GSEA analysis on Community 1 in DEG value ranking. **E** Identification of Community 1-related immune genes in multiple HNSCC cohorts. **F** Umap plots show immune cell subsets identified through the ProjecTILs algorithm in annotated T cells. **G** Distribution of immune cell subtypes in different conditions. **H** Diffusemap shows the distribution of cells in different immune subsets and conditions in dynamic evolution trajectories of CD8 T cells. **I** Violin plot shows DEGs in CD8 T cells of MOC1 and MOC1esc1. **J** KEGG analysis of DEGs in CD8 T cells of MOC1 and MOC1esc1. **K** Diffuse map shows dynamic evolution trajectories of MOC1 CD8 T cells. **L** Changes in TCR clone size before and after ICI treatment in the dynamic evolution trajectories of MOC1 CD8 T cells. **M**, **N** Violin plot and Line plot show DEGs in MOC1 CD8 T cells before and after ICI treatment and their changes in the dynamic evolution trajectories of CD8 T cells. **O** The identification of iks. **P** Diffuse map shows the distribution of iks in the dynamic evolution trajectories of CD8 T cells. **Q** Jaccard similarity coefficient between iks and ProjecTILs immune cell subtypes. **R** The hill score of iks. **S** Identification of cell–cell pairs related to Community 1-related immune genes in CellPhoneDB. **T** Differential cell–cell pairs in Community 1-related immune genes between MOC1(response) and MOC1esc1(resistant)

To investigate the immune cells in TME with GSE153383 dataset, MOC1(anti-PD1: response) and MOC1esc1 (anti-PD1: resistant) cell lines were applied to DEG analysis (Fig. 7A, See details in Additional file 3: Method 4, 5). 17 genes in Community 1 (threshold of abs (log2FoldChange) > 0.5, FDR < 0.05) were significantly elevated in MOC1, while only 2 genes were significantly elevated in MOC1esc1 (Fig. 7B). Compared with the non-Community 1 genes, Community 1 contained a significantly higher proportion of DEGs (Chi-square test *p* = 2.3e-10) (Fig. 7C). The GSEA analysis also showed that Community 1 was significantly enriched (NES = 1.5,* p* = 4.5e-4) in the ranked difference of gene expression between MOC1 and MOC1esc1 (Fig. 7D). These results suggested that Community 1 was closely related to OSCC responses to anti-PD1 therapy. Then, this relation was analyzed at single-cell level. First, 497 Community 1-related immune genes were identified through multiple HNSCC cohorts (See Materials and Methods for details. Fig. 7E). 16,885 qualified cells selected from the scRNA-seq data of MOC1 and MOC1esc1 were annotated into 8 clusters, including B cells (Cd19, Cd79a and Cd79b), CD4 T cells (Cd4), CD8 T cells (Cd8a), DC (Flt3 and Ccl9), TAMM (M1 like Ccl2, Ccl9; M2 like Arg1 and Mrc1), Neutrophils (S100a9 and S100a8), Nk (Ncr1 and Nlrb1c) and Treg (Foxp3) (Additional file 1: Fig. S9A, B). The cell distribution in the four conditions (MOC1, MOC1esc, treatment and control) was shown in Additional file 1: Fig. S9C. Because T cells in immune TME were too complex and heterogeneous to be categorized by the traditional markers, we used the ProjecTILs algorithm to project the annotated T cells (CD8 T, CD4 T and Treg) into the referenced single-cell atlases, through which T cells were divided into 9 subpopulations (CD8_Tex, CD8_Tpex, CD8_EffectorMemory, CD8_EarlyActiv, CD8_NaiveLike, CD4_NaiveLike, Tfh, Th1 and Treg) (Fig. 7F). The T cells in MOC1, in which CD8_Tex was mainly distributed in MOC1 treatment group, were much more than those in MOC1esc1 (Fig. 7G). Because of the pivotal role of CD8 T cells in ICI, we used diffusion maps to visualize the dynamic relationship among CD8 T subpopulations. The 2D diffusion trajectory maps divided T cells into branch 1 mainly in MOC1, and branch 2 mainly in MOC1esc1. In both branches, CD8_NaiveLike and CD8_EarlyActiv were located at the beginning, CD8_EffectorMemory and CD8_Tpex in the middle, and CD8_Tex at the distal end (Fig. 7H). All these findings suggested that the similar changing tendency of CD8 T subtypes in different ICI resulted from different transcriptomic profiles.

We found the immune exhaustion in the CD8 T cells in MOC1, such as the high expression of Ctla4 and Tgfb1, and the faint expression of Ifng (F ig. 7I). The enrichment analysis displayed the signatures of both immune activation and suppression in MOC1. In contrast, the transcriptomic features of MOC1esc tended to the slight immune activation (Fig. 7J). Then, we further explored the alterations in transcriptome and T-cell receptor (TCR) of MOC1 CD8 T cells before and after anti-PD1 immunotherapy. We used diffusion map to visualize the 2D trajectory of CD8 T cells in the MOC1 group, in which the control group was mainly located at one end of the trajectory, while the treated group was diffusely distributed throughout the trajectory (Fig. 7K). The scTCR-seq analysis showed that the clone numbers of treated group were significantly larger than those of control group in the whole trajectory (Fig. 7L). We visualized several differential markers between control and treated groups in CD8 T cells in MOC1 (Fig. 7M), as well as their changes on the trajectory (Fig. 7N), which suggested that anti-PD1 responses not only enhanced the TCR diversity of MOC1 CD8 T cells, but also drove their transcriptomic features into a mixture of immune hot (Cd8a and Ccl5) and cold (Pdcd1 and Lag3), verifying the contradictoriness and complexity in the responses to anti-PD1 in TME.

To reveal the relationship between Community 1 and anti-PD1 responses in TME, we obtained 87 genes by intersecting Community 1-related immune genes into the first 1000 genes of CD8 T-cell hypervariable genes. Then, the top 30 genes were extracted for kmeans clustering of CD8 T cells to produce 8 clusters (ik1-ik8, immune-related Kmeans clusters abbreviated as ik) (Fig. 7O) which distributed on the diffuse map (Fig. 7P). The Jaccard similarity coefficient of the 8 clusters with the subtypes of ProjecTILs was calculated, and found that ik4 (Tnfsf11 and Hif1a), ik7 (Cdk6 and Havcr2) and ik6 (Itgb1) exhibited the high similarity with CD8_Tpex, CD8_Tex (the CD8 T subtype most related to anti-PD1) and CD8_EeffectorMemory, respectively (Fig. 7Q), suggesting that Community 1 mediated the anti-PD1-related CD8 T exhaustion transcription signature in TME (ik4 and ik6), which was accompanied by the significantly reduced TCR diversity (Fig. 7R). Finally, in 497 Community 1-related immune genes, 249 annotated Cell–Cell communication pairs were identified from CellPhoneDB database (Fig. 7S), among which the most significantly differential expression pairs between anti-PD1 responsive and resistant subgroups (CCL5-CCR1, TGFB1-TGFBR3, CTLA4-CD86, etc.) were selected and visualized (Fig. 7T). These results suggested that Community 1 mediated anti-PD1-related cell communications in TME.

### BHG linked upstream IWHMB with downstream tumor progression and immune-related transcriptomic alterations in biological networks

**Fig. 8:**
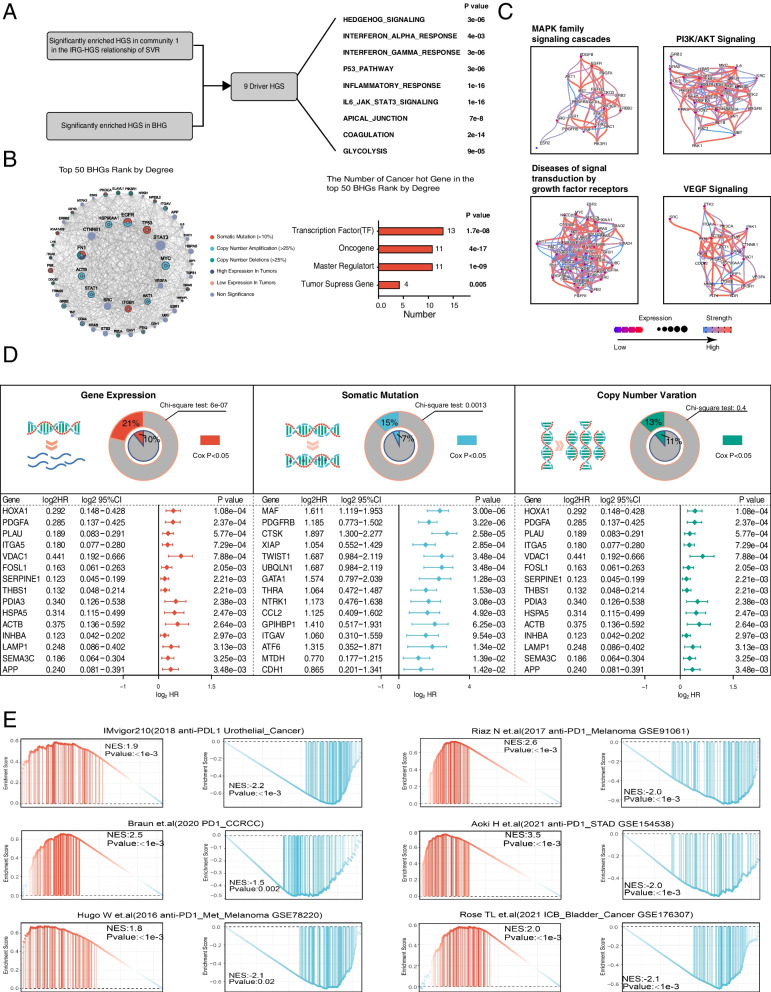
Identification and analysis of BHG **(A)** Identification of Driver HGS. **B** Proportion of different cancer Hot genes in BHG. **C** Bayesian network visualization of 4 cancer driver pathways enriched in BHG. **D** Forest plots showing the top 15 genes with significant pvalue for BHG in the Cox model at 3 omics levels. **E** GSEA analysis of BHGs in 6 ICI cohorts (response vs. resistant)

We have thus resolved how IWHMB was associated with HNSCC progression phenotype in a data-driven model. Then, we integrated global network to elucidate the biological association of IWHMB with HNSCC progression phenotype. Based on the Oncotecture hypothesis [15, 18], we hypothesized two characteristics of the specific Tumor Checkpoints: 1) in a specific cancer pathway or biological pathway; 2) linking genes to both cancer progression phenotype (Community 1) and cancer immune phenotype (Community 1-related immune genes). We have named MRs with these characteristics after Bridging hub genes (BHGs). The BHGs were searched in Community 1 and Community 1-related genes, respectively, and also worked as seed nodes in global networks (from three PPI: STRING (interaction score > 700), BIOGRID and KEGG; TF-Targert: Constructed from GSE103322 dataset using SCENIC). Then, node prioritization was performed using RWR algorithm (See details in Additional file 3: Method 3) to extract the top 500 genes in the descending order of their scores. Finally, 249 BHGs were obtained by intersecting the two sets of top 500 genes (Additional file 2: Table S7). To confirm whether BHGs are present in specific upstream signaling pathways or biological pathways (Driver HGS), we obtained 12 HGSs significantly enriched in Community 1 based on the established relationship between Gene expression and HGS perturbation in SVR. All the 12 HGSs drove Community 1 expression in a data-dependent manner. 9 of the 12 HGSs were significantly enriched in BHGs. The 9 HGSs have not only data correlation, but also network correlation on the driver of Community 1. Therefore, the 9 HGSs were considered as Driver HGSs (Fig. 8A). To further analyze the BHG features, we visualized the Hub Genes (top 50 in Degree ranking) in 249 BHGs, most of which had genomic variants (Fig. 8B). Meanwhile, since BHG was enriched in cancer driver and progression-related pathways (Additional file  2: Table S8), we constructed and visualized the regulatory network of BHGs in the 4 driver pathways (PI3K/AKT Signaling in Cancer, MAPK family signaling cascades, Diseases of signal transduction by growth factor receptors and Signalings by VEGF) based on gene expression data and clinical information (tumor/normal) using CBNplot (Fig. 8C).

We examined the association of BHG with prognosis and ICI response using TCGA HNSCC cohort and ICI cohorts. At the transcriptomic and genomic levels, BHG contained a significantly higher proportion of prognostic genes than non-BHG. The forest plot showed the top 15 genes with significant Cox model pvalues at 3 omics levels (Fig. 8D). According to the differential expression of BHGs between Response (PR, CR) and Resistant (SD, PD) subgroups in ICI cohorts, the BHGs were divided into Response BHGs and Resistant BHGs for GSEA analysis. The significant enrichment of BHGs in the ranked gene difference of the 6 ICI cohorts suggested a close association of BHGs with ICI responses (Fig. 8E). Above findings indicated that BHGs represent a general gene group, most of which were located in the hubs of tumor pathway networks. Thus, even the transcriptome was regarded as the most direct reflector, the mutations in BHGs (including genomic alterations, CNV and epigenetic changes) would impact the core of tumor networks more directly.

### BHG outperformed other predictive gene signatures via the high stability and robustness in predicting ICI responses

**Fig. 9:**
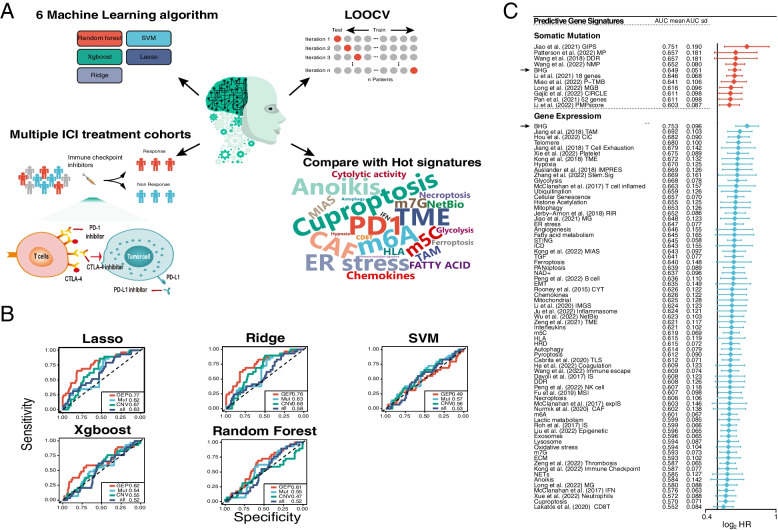
Comparison of BHG and other predictive gene signatures in multiple ICI cohorts **(A)** Overview of the BHG-based ICI prediction model. **B** AUC values for predicting ICI responses in the Braun et al. (2020) cohort by combining 6 machine learning models based on 3 omics of BHG. **C** Comparison of AUC means of BHG with 10 and 70 gene signatures in multiple ICI cohorts for predicting ICI response at the somatic mutation and gene expression levels, respectively

To demonstrate the pivotal role of BHG in cancer progression and immune networks, we tested the predictive capacity of BHG on ICI response in 10 independent ICI cohorts (Fig. 9A See details in Additional file 3: Method 6). We tested 5 machine learning methods in Braun et al. (2020) cohort, each using 3 omics of the BHG (Gene Expression Profile: GEP, Single Nucleotide Variants: SNV, Copy Number Variants: CNV) as predictor variables, it was found that the prediction performance of the GEP-based model was significantly higher than those of the other two omics, and the prediction performance of the logistic regression model with penalty terms was significantly higher than those of the other models among the different machine learning methods (Fig. 9B). Thus, lasso regression was used as a machine learning model to test the efficacy of BHG and other public gene signatures in predicting ICI responses. At the somatic mutation level, BHG was compared with 10 public gene signatures (Additional file 2: Table S9) in 5 independent ICI cohorts, and BHG achieved a mean predictive value of 6.5 AUC (5th place). At the gene expression level, BHG was compared with 70 public gene signatures (Additional file 2: Table S10) in 10 independent ICI cohorts, and BHG achieved a 7.5 mean AUC (1th place) significantly higher than other public gene signatures (Fig. 9C). Additional file 1: Fig. S10A shows the predictive capacity of BHG and 10 gene signatures at the somatic mutation level in 5 independent ICI cohorts. Additional file 1: Fig. S10B shows the predictive power of BHG and 70 gene signatures at the gene expression level in 10 independent ICI cohorts.

## Discussion

In HNSCC, the increasing malignancy and immune escape mediated by EMT directly result in poor prognosis and ICI resistance. Within the genome-transcriptome-phenotype framework under the functional genomics of cancer, we explored the driving force on EMT and immune escape in the perspective of pathway mutations, and developed the unique biomarkers to predict prognosis and ICI response.

### IWHMB captures pathway mutational features at the individual level and eliminates global TMB interference

Hierarchical clustering based on IWHMB divided both HNSCC cohorts into 12 clusters, of which the somatic mutations were enriched in specific HGS. This result indicated that IWHMB not only eliminated TMB interference, but also captured the individual specific mutated pathway signature in large cohorts. On this basis, we depicted the atlas of pathway mutations in HNSCC and identified 12 pathway mutation-associated subtypes. We found that the subtypes enriched for protein secretion-associated mutations have low TMB and high CNV load, suggesting a potential link between the protein secretion pathway and CNV. A lot of studies demonstrated the promotion of Hedgehog signaling on tumor stromal components [38]. The C1 cluster characterized by HH signaling mutations displayed the high stromal score and mesenchymal subtype in Kech classification [35]. The C7 cluster with the high immune score enriched the mutations related to interferon response, which verified the association of mutation in interferon-related pathway with immune activation. Extracellular vesicles (EV) are closely associated with ECM, since the ECM-related gene mutations often impact the production and transportation of EV [39]. Our study indicated that the transcriptomic alterations in the C5 cluster enriching the mutations in EMT-related genes showed a robust correlation to EV-related processes.

### IWHMB score correlated the status of pathway mutations with HNSCC clinical phenotype

IWHMB could distinguish HPV positive patients from negative group because the somatic mutations of TP53 and CDKN2A almost only present in HPV negative HNSCC patients [40, 41]. Thus, the IWHMB of P53_PATHWAY and DNA_REPAIR, in whichTP53 and CDKN2A acted as key knots, was significantly elevated in HPV negative group. IWHMB could also distinguish pharyngeal squamous cell carcinoma from HNSCC. Clinically, pharyngeal squamous cell carcinoma prone to a worse prognosis because of the higher tendency of infiltration and metastasis. The IWHMB indicated that the somatic mutations of pharyngeal squamous cell carcinoma were significantly enriched in the pathways related to EMT and metastasis. Moreover, the recent study reported that the somatic mutation of CASP8, a gene regulating apoptosis, only took place in oral squamous cell carcinoma, but almost not in pharyngeal squamous cell carcinoma [34], which was confirmed by the higher IWHMB of APOPTOSIS in oral squamous cell carcinoma than that in pharyngeal squamous cell carcinoma. IWHMB was able to distinguish the clinical stages and metastasis of HNSCC, which was applied to predict core factors of prognosis, such as the imbalance of cell cycle regulation and EMT. In the IWHMB score system, the mutations of cell cycle and EMT usually suggested a worse prognosis, even the metastasis in late stage. Additionally, we also found a pathway, sterol metabolism, correlated with tobacco and alcohol consumption. The mutations in sterol metabolism pathway were correlated with HNSCC prognosis, which has been verified by several studies [42, 43]. Eventually, according to IWHMB, the cancer classification of all 32 kinds of TCGA tumors is correlated to prognosis at more or less extent, verifying the capability of IWHMB classification in identifying the prognostic subtype of tumors.

### Transcriptomic alterations will cluster and reveal the association of IWHMB with cancer phenotype

Studies using multiple cohorts demonstrate that although the somatic mutations vary in different tumor patients, their transcriptome share a great similarity [44]. Our multiple regression models indicated the changes in individual gene expression was often perturbed by the mutations in multiple pathways or biological processes. Such perturbation relationship is conserved in both HNSCC cohorts, suggesting that transcriptomic features were more reliable in revealing the association of PMB with tumor phenotype.

Complex biological networks consisting of a large number of nodes and edges are hard to be interpreted holistically. Therefore, to explore the transcriptomic alterations associated with tumor phenotype, we have to dissociate the complicated network into communities. The five communities identified by ARVGA algorithm are associated with 4 HNSCC phenotype in below aspects: 1). Promoting EMT through metabolism and remodeling of extracellular matrix components; 2). Driving tumor cell growth and metabolism by affecting energy metabolism and ribosomal processes [37]; 3). Involving in antiviral and genomic responses to virus; 4). Contribution to the de-differentiation and stemness of HNSCC cells through gene transcription related to craniomaxillofacial development [45].

### ECM-related Community 1 most likely acts as the core component affecting HNSCC progression phenotype

As a key non-cellular component in TME, ECM not only provides the scaffold for the adhesion and migration of tumor cells, but also mediates the interactions between tumor cells and TME. In addition, ECM also regulates tumor-specific behaviors, such as anti-apoptosis, infinite proliferation, blood vessel invasion, metastasis, etc. [46]. This was confirmed in a series of data-driven studies recently, in which the Community 1 rich in ECM-related genes was robustly associated with clinical prognosis, clinical stage, and distant metastatic status in multiple HNSCC cohorts. Latest study demonstrated that abnormal extracellular matrix dynamically promotes the conversion of stem cell niche into a cancerous one [47], which is also verified by the positive correlation between Community 1 and stemness of tumor cells in our study. More importantly, ECM plays the regulatory roles in multiple stages of tumor immune cycle, for examples, the rigorous ECM inhibits apoptosis and release of tumor antigens; ECM regulates the activation, migration and elimination of tumor cells by T cells [48]. Multiple cohorts in our study suggested the varying extent of correlation between ECM-related Community 1 and immune score. The notion in the latest study proposed that tumor rebuilds ECM and releases the components into circulating blood, which could work as the hallmarks for tumor diagnosis or prognosis. Our study improved this notion from the perspectives of data and network. ECM-related genes as co-expression network modules (Community 1) showed strong stability and highly variable transcription in multiple HNSCC cohorts, which enforces the potential of ECM as tumor hallmarks. Taken together, ECM-related Community 1 is the key community connecting PMB and HNSCC progression phenotype.

### Single cell omics reveals the association of Community 1 with tumor progression and immune

First, we found that the genes in Community 1 promoted the EMT of HNSCC. In the pseudotime trajectory of tumor EMT, the increasing tendency of the average gene expression in Community 1 was highly coincided to EMT trajectory. The GSVA scores in the trajectory of Community 1 were highly positive associated not only with the GSVA scores of EMT-related signatures, but also with those of tumor progression-related processes. Above findings suggested the intensive promotion by the increasing expression of Community 1 on the EMT and progression of HNSCC. Furthermore, the distribution of Community1-based clusters on the pseudotime trajectory present how the genes in Community 1 participated in EMT. The ek1-ek4 sharing the similar expression pattern were distributed in the early stage of the trajectory, and their hallmark genes were correlated with the early features of tumor EMT (such as cell adhesion: GJA1 [49], stemness: DKK3 [50], CD276 [51], and ECM regulation: PLAU, PDPN and SERPINE1 [52–54]), which suggested the roles of Community 1 in early EMT. TP63, a transcription factor related to HNSCC initiation was highly enriched in ek1, ek 2 and ek 4. Particularly, ek2 is distinguished in transcription regulation and biological function, because it enriched FKBP10, a promoter for tumor proliferation and invasion through the crosstalk to PIK3 pathway, BCL11A and MSC, the tumor stemness-related transcription factors [51, 55]. Since BCL11A also plays a critical role in the differentiation of immune cells [56], ek2 enriches the immune activation-related pathways. In contrast, ek5-ek8 encode ECM-related proteins, which are the majority of Community 1. Ek7 highly enriches TAGLN (a smooth muscle marker), smooth muscle-related transcription factors (LMO2 and KLF2), as well as smooth muscle-related pathway in Community 1. Ek6 and ek8 encode the main components of stromal cells, and highly enrich late EMT features, such as the transcription factors (RUNX2 and TWIST2) and ECM-related processes. The above results demonstrated how genes in Community 1 are involved in and regulate the dynamic evolution of HNSCC EMT. Second, the genes in Community 1-related immune genes modify the pattern of ICI responses in TME of OSCC. By deep mining the single cell multi-omics from the mouse models response/resistant to anti-PD1 therapy, the GSEA analysis revealed that Community 1 was enriched in the OSCC mice sensitive to anti-PD1 therapy, implicating the correlation between Community 1 and ICI responses in OSCC. ICI responses were correlated with the subtypes of CD8 T cells. In the OSCC mice receiving anti-PD1 therapy, although the dynamic trajectories of CD8 T cells were similar in different conditions, the percentages of subtypes were significantly heterogeneous. Most evidently, the ICI sensitive group contained more exhaustive CD8 T cells than other groups, suggesting a close correlation of the exhaustive CD8 T cells to ICI responses [57]. Community 1 is able to regulate the subtypes of CD8 T cells related to ICI responses, in particular, the exhaustive CD8 T cells. In Community 1-related immune genes, Cdk6 and Havcr2 are robustly expressed in the exhaustive CD8 T cells, playing roles in connecting EMT and tumor immune. CDK6 regulates TGFβ1-induced EMT via epigenetic mechanism [58] and immune surveillance by decreasing PD-L1 stability [59]. As the marker of exhaustive CD8 T cells, HAVCR2 regulates EMT by crosstalking Akt/GSK-3β/Snail signaling pathway [59] with SMAD7/SMAD2/ SNAIL1 Axis [60], implicating the dual role of Community 1-related immune genes in EMT and tumor immune. In addition, Community 1 regulates intercellular communication associated with ICI responses. Some Community 1-related genes, such as CTLA4_CD86, which are common immune checkpoints with their expression significantly correlated with ICI responses; TGFB1-TGFBR3 and TGF pathway, which have been strongly associated with immune escape and the activity as a marker of ICI responses; Chemokine CCL4, which is highly expressed in effector memory T cells of ICI-sensitive patients.

### Network science confirms the pivotal role of BHGs in cancer biological networks, which converges upstream specific signaling pathway mutation loads and initiates downstream transcriptomic alterations affecting cancer phenotype

Since based on Tumor Checkpoint, the concept of BHG inherits the characteristics of Tumor Checkpoint. The activation of Tumor Checkpoint originates from the alteration of upstream signals. In this study, BHG was significantly enriched in the upstream 9 Driver HGS that were highly correlated with Community 1 expression, which corresponds to the activation of BHG by IWHMB in 9 Driver HGS. Secondly, Tumor Checkpoint regulated downstream transcriptomic alterations are closely related to cancer phenotype. The BHGs were identified based on cancer progression and immune phenotype-related genes using random walk restart algorithm, so they are not only adjacent to cancer phenotype-related nodes in the biological network, but also in the hub position, which indicates that the alteration of BHGs could perturb the whole cancer biological network. BHG not only has the characteristics of Tumor Checkpoint, but also breaks some limitations of Tumor Checkpoint. Firstly, Tumor Checkpoint overemphasizes the influence of transcriptional regulatory networks and thus, is limited to transcriptomic factors [61, 62]. BHG extends the concept of Tumor Checkpoint to the whole biological network, so the genes constituting BHG are not restricted to transcriptional factors only, but include a variety of oncogenes and TSG. Secondly, since the identification of MR started from upstream genomic alterations, it was difficult to explore the relationship between MR and cancer transcriptomic alterations though the strategy clarifying the relationship between upstream mutations and MR. Instead, we first identified the transcriptomic features closely related to HNSCC phenotype and then, identified the BHG. In this way, the relationship between BHG and HNSCC transcriptome would be explicated. To demonstrate this close relationship, we used prediction of ICI response as a validation index. The predictive ability to ICI response not only represents the robustness of biomarker, but also reflects its capability of capturing the transcriptomic alterations of Transcriptomic changes associated with resistance or response to ICI treatment. The reason BHG can predict ICI response mainly comes from two aspects: 1. By activating the IFN $\gamma$-JAK-STAT pathway to create a hot tumor microenvironment to recruit T cells [63, 64]. 2. Through the expression of genes in Oncogenic pathways such as TP53, EGFR, CTNNB1, etc., to maintain Oncogenic signaling, thereby forming a tumor immune-suppressive microenvironment to promote immune escape [40, 65]. Other gene signatures, such as T cell inflamed, Chemokines, Interleukins, etc. only represent a part of tumor hot transcriptional signatures; DDR and STING are only parts of tumor hot immunity related pathways; while CAF, TAM and Immune escape signatures only reflect the escape phase of tumor immunity. In conclusion, these gene signatures only partially portray tumor immunity, instead of globally delineating the hot transcriptional features at tumor immune network, so the robustness of prediction models based on them is obviously inferior to that of BHG.

## Conclusions

PMB algorithm was improved by integrating pathway structure information and eliminating the interference of global TMB to better respond to the patient's pathway mutation status. Multiple algorithmic models were then used to reveal the drive of pathway mutations on EMT and immune escape in HNSCC. Finally, the unique biomarkers for predicting prognosis and ICI response were identified.

### Supplementary Information

**Additional file 1:** **Fig. S1**. The relationship between IWHMB and clinical stage, metastasis, smoking, and drinking in the TCGA HNSCC and Chen, H et al. 2021 cohort. (A) HGS with high IWHMB scores in early-stage clinical patients. (B) HGS with high IWHMB scores in late-stage clinical patients. (C) HGS with high IWHMB scores in patients with metastasis. (D) HGS with high IWHMB scores in tobacco-using patients. (E) HGS with high IWHMB scores in patients who consume alcohol. **Fig. S2**. The relationship between the IWHMB for three types of EGFR signaling and prognosis, clinical stage, and metastasis in the TCGA HNSCC and Chen, H et al. 2021 cohort. (A) Venn diagram of gene relationships in the three EGFR pathways. (B) IWHMB scoring of the three types of EGFR signaling in different HPV statuses. (C) The relationship between the IWHMB for three EGFR pathways and prognosis in the TCGA HNSCC Negative HPV, Positive HPV, and the Chen cohort. (D) The relationship between the IWHMB for the three EGFR pathways and clinical staging in the TCGA HNSCC and Chen cohorts, as well as the metastatic status in the TCGA HNSCC cohort. **Fig. S3**. Multiomics differences in IWHMB-associated cancer subtypes in Chen, H et al. 2021 cohort. (A) Circular cluster dendrogram showing 12 IWHMB-associated cancer subtypes. (B) Heatmap showing 12 IWHMB-associated cancer subtypes. (C) Clinical prognosis of 12 IWHMB-associated cancer sub-types. (D) Somatic mutation waterfall plot of 12 IWHMB-associated cancer subtypes. (E) Differential copy number changes (Fisher's precision probability test pvalue <0.05) in 12 IWHMB-associated cancer subtypes. (F) TMB of 12 IWHMB-associated cancer subtypes. (G) CNV Burden of 12 IWHMB-associated cancer subtypes. (H-J) StromalScore, TumorPurity and ImmuneScore of 12 IWHMB-associated cancer subtypes. (K) Relationship between IWHMB-associated cancer subtypes and Kech subtypes. (L) DEGs of 12 IWHMB-associated cancer subtypes. (M) GSEA pathway enrichment of 12 IWHMB-associated cancer subtypes. **Fig. S4**. Relationship between IWHMB-related subtypes and clinical prognosis in 32 TCGA cancers. **Fig. S5**. Mutation Signatures in TCGA HNSCC cohort. (A) NMF of mutation signatures in the TCGA HNSCC cohort. (B) Cosine similarity of mutation signatures in the TCGA HNSCC cohort with annotated signatures recorded in the COSMIC database. (C) Association of 6 mutational signatures with IWHMB-related subtypes in the TCGA HNSCC cohort. **Fig. S6**. ARGVA algorithm is used to identify Communities of IRG. (A) Schematic diagram of ARVGA algorithm. (B) Iteration period and loss function of ARVGA algorithm. (C) Communities visualization. (D) Interaction plot of 50 HGS disturbed genes. (E) Interaction plot of 50 HGS disturbed genes with the HGS itself. **Fig. S7**. Network properties of Communities. (A) GO and KEGG enrichment analysis of Communities. (B) The CRISPR-based or RNAi-based gene dependency scores between Communities. (C) Average variability of Communities gene expression across multiple HNSCC cohorts. (D) Network similarity of Communities in multiple HNSCC cohorts. (E) Community 1 network visualization. Different colors represent that gene is interfered by different IWHMB of HGS. (F) Enriched path visualization in Community 1. **Fig. S8**. Single cell annotation of GSE103322. (A, B) UMAP plot of GSE103322 single cells, colors represent tissue origin and cell type respectively. (C) Expression of marker genes in different cell types. (D) Kmean clustering of malignant epithelial, fibroblastic, and endothelial cells in GSE103322 using gene expression in community 1. **Fig. S9**. Single cell annotation of GSE153383. (A) UMAP plot of GSE153383 single cells, colors represent cell type. (B) Expression of marker genes in different cell types. (C) Distribution of GSE153383 cells on UMAP plots under different conditions. **Fig. S10**. Comparison of AUC values of BHG with other public gene signatures. (A) Comparison of AUC values of BHG with 10 gene signatures at the somatic mutation level for predicting ICI response in 5 ICI cohorts. (B) Comparison of AUC values of BHG with 70 gene signatures at the gene expression level for predicting ICI response in 10 ICI cohorts.

**Additional file 2:** **Table S1**. Clinical characteristics of the HNSC patients used in this study.

**Additional file 3:** **Supplementary methods**.

## Data Availability

The data used to support the fndings of this study are available from public databases, including TCGA (pan-cancer, TCGA-HNSCC and chen’s HNSCC cohort) database (https://portal.gdc.cancer.gov/), Cancer Dependency Map (HNSCC cell gene dependency data) database (https://depmap.org/), GEO (HNSCC: GSE65858, GSE39366, GSE40774, GSE41613, and GSE117973. Single cell omic data: GSE103322 and GSE153383. ICI cohort: GSE78220, GSE91061, GSE154538, GSE176307, GSE93157, GSE100797 and GSE126044) database (https://www.ncbi.nlm.nih.gov/geo/). Other ICI treatment cohorts are acquired as follows: IMvigor210 (2018 anti PDL1 Urothelial_Cancer, http://research-pub.gene.com/IMvigor210CoreBiologies/packageVersions/) [19], Braun et al. (2020 PD1_CCRCC, https://www.nature.com/articles/s41591-020-0839-y) [20], Liu et al. (2019 anti PD1 Met Melanoma, https://www.nature.com/articles/s41591-019-0654-5) [25], Gide et al. (2019 anti PD1 + CTLA4 Melanoma, http://tide.dfci.harvard.edu/) [27], Nathanson et al. (2017 anti CTLA4 Melanoma http://www.hammerlab.org/melanoma-reanalysis) [29]. The code used to support the findings of this study are available from the corresponding author upon request.

## Abbreviations

BHG: Bridge Hub Gene
HNSCC: Head and neck squamous cell carcinoma
CNV: Copy Number Variation
IWHB: Individualized Weighted HGS Mutation Burden
DEG: Differentially expressed genes
ICI: Immune checkpoint inhibitor
DepMap: Dependency Map
SNV: Single Nucleotide Variation
ECM: Extracellular Matrix
SVR: Support vector machine regression
EMT: Epithelial Mesenchymal Transition
TCGA: The Cancer Genome Atlas
GEO: Gene Expression Omnibus
TCR: T-cell receptor
HGS: Hallmark Gene Set
TMB: Tumor mutation burden
