# Supplementary material for: Quantified pathway mutations associate epithelial-mesenchymal transition and immune escape with poor prognosis and immunotherapy resistance of head and neck squamous cell carcinoma
Source: BMC Med Genomics. 2024 Feb 8;17:49. doi: 10.1186/s12920-024-01818-6 (PMC10854145; doi:10.1186/s12920-024-01818-6)
Supplement: Supplementary file 1 — Additional file 1: Fig. S1. The relationship between IWHMB and clinical stage, metastasis, smoking, and drinking in the TCGA HNSCC and Chen, H et al. 2021 cohort. (A) HGS with high IWHMB scores in early-stage clinical patients. (B) HGS with high IWHMB scores in late-stage clinical patients. (C) HGS with high IWHMB scores in patients with metastasis. (D) HGS with high IWHMB scores in tobacco-using patients. (E) HGS with high IWHMB scores in patients who consume alcohol. Fig. S2. The relationship between the IWHMB for three types of EGFR signaling and prognosis, clinical stage, and metastasis in the TCGA HNSCC and Chen, H et al. 2021 cohort. (A) Venn diagram of gene relationships in the three EGFR pathways. (B) IWHMB scoring of the three types of EGFR signaling in different HPV statuses. (C) The relationship between the IWHMB for three EGFR pathways and prognosis in the TCGA HNSCC Negative HPV, Positive HPV, and the Chen cohort. (D) The relationship between the IWHMB for the three EGFR pathways and clinical staging in the TCGA HNSCC and Chen cohorts, as well as the metastatic status in the TCGA HNSCC cohort. Fig. S3. Multiomics differences in IWHMB-associated cancer subtypes in Chen, H et al. 2021 cohort. (A) Circular cluster dendrogram showing 12 IWHMB-associated cancer subtypes. (B) Heatmap showing 12 IWHMB-associated cancer subtypes. (C) Clinical prognosis of 12 IWHMB-associated cancer sub-types. (D) Somatic mutation waterfall plot of 12 IWHMB-associated cancer subtypes. (E) Differential copy number changes (Fisher's precision probability test pvalue <0.05) in 12 IWHMB-associated cancer subtypes. (F) TMB of 12 IWHMB-associated cancer subtypes. (G) CNV Burden of 12 IWHMB-associated cancer subtypes. (H-J) StromalScore, TumorPurity and ImmuneScore of 12 IWHMB-associated cancer subtypes. (K) Relationship between IWHMB-associated cancer subtypes and Kech subtypes. (L) DEGs of 12 IWHMB-associated cancer subtypes. (M) GSEA pathway enrichment of 12 IWHMB-associated cancer [file 12920_2024_1818_MOESM1_ESM.pdf]

Supplementary Figures

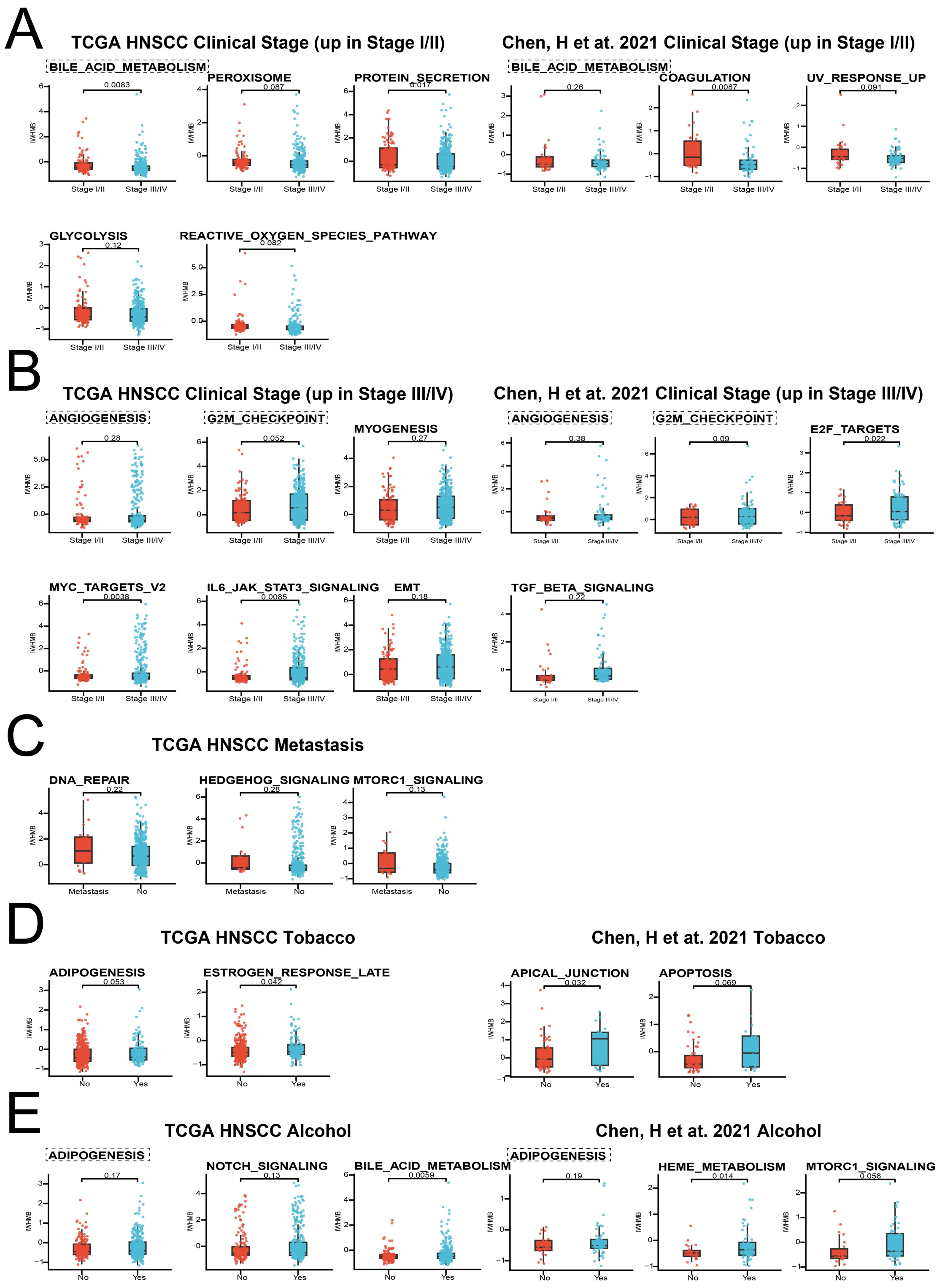

Fig. S1. The relationship between IWHMB and clinical stage, metastasis, smoking, and drinking in the TCGA HNSCC and Chen, H et al. 2021 cohort. **(A)** HGS with high IWHMB scores in

early-stage clinical patients. **(B)** HGS with high IWHMB scores in late-stage clinical patients. **(C)** HGS with high IWHMB scores in patients with metastasis. **(D)** HGS with high IWHMB scores in tobacco-using patients. **(E)** HGS with high IWHMB scores in patients who consume alcohol.

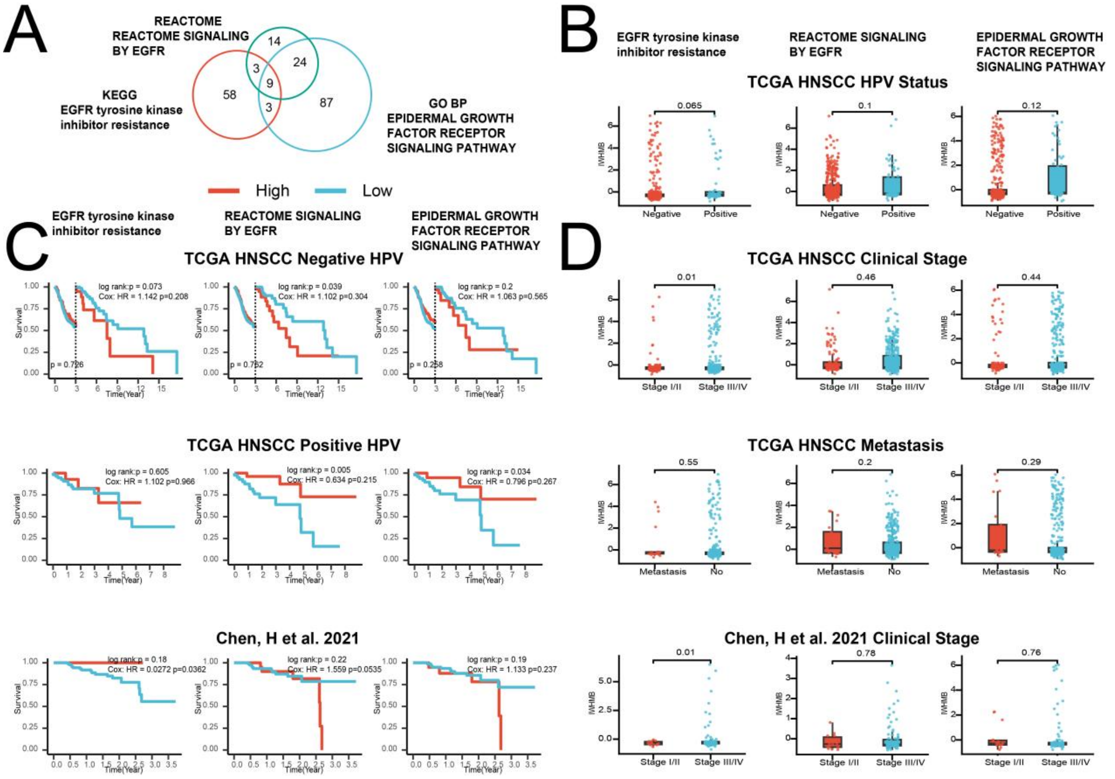

Fig. S2. The relationship between the IWHMB for three types of EGFR signaling and prognosis, clinical stage, and metastasis in the TCGA HNSCC and Chen, H et al. 2021 cohort. **(A)** Venn diagram of gene relationships in the three EGFR pathways. **(B)** IWHMB scoring of the three types of EGFR signaling in different HPV statuses. **(C)** The relationship between the IWHMB for three EGFR pathways and prognosis in the TCGA HNSCC Negative HPV, Positive HPV, and the Chen cohort. **(D)** The relationship between the IWHMB for the three EGFR pathways and clinical staging in the TCGA HNSCC and Chen cohorts, as well as the metastatic status in the TCGA HNSCC cohort.

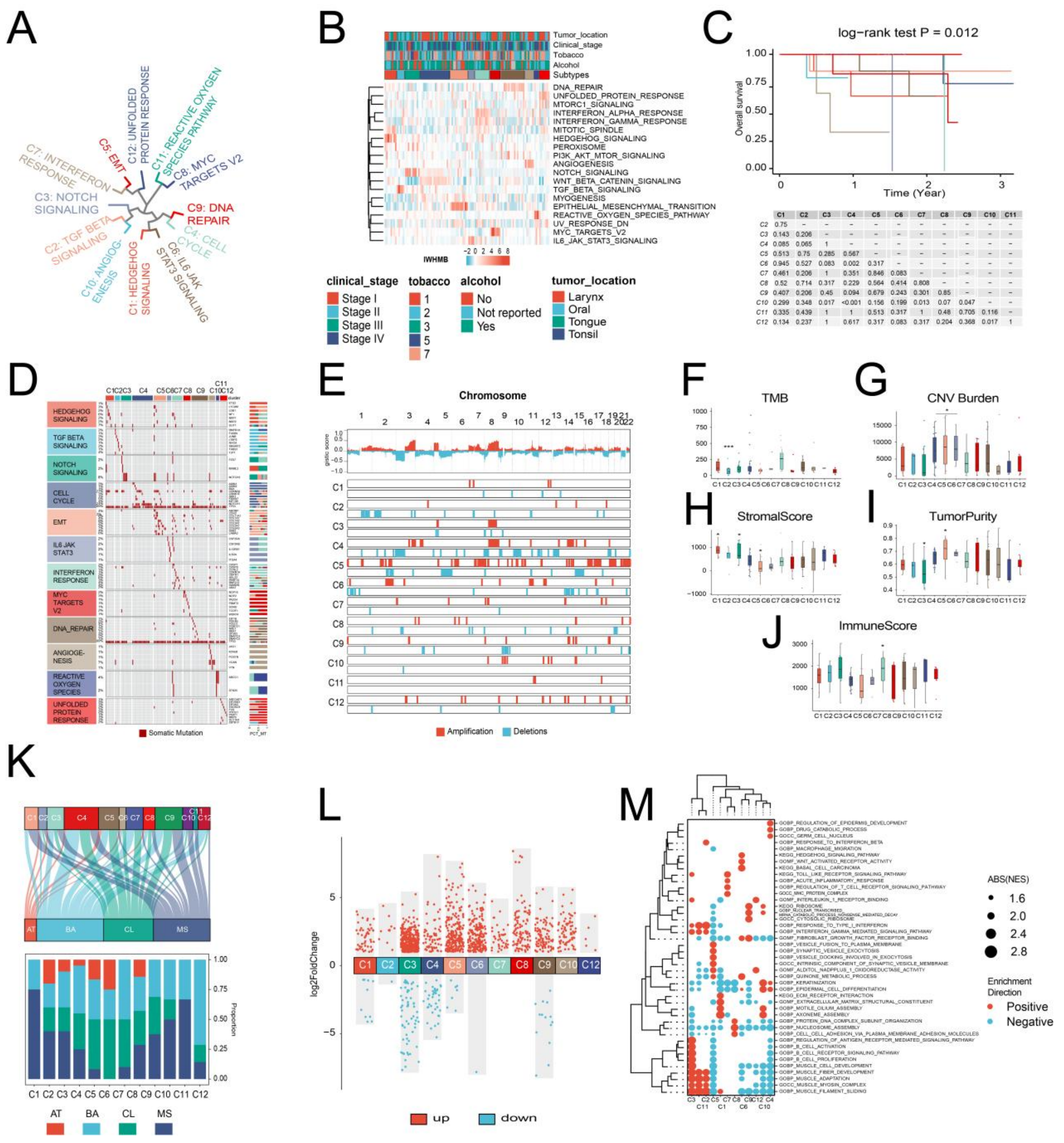

Fig. S3. Multiomics differences in IWHMB-associated cancer subtypes in Chen, H et al. 2021 cohort. **(A)** Circular cluster dendrogram showing 12 IWHMB-associated cancer subtypes. **(B)** Heatmap showing 12 IWHMB-associated cancer subtypes. **(C)** Clinical prognosis of 12 IWHMB-associated cancer sub-types. **(D)** Somatic mutation waterfall plot of 12 IWHMB-associated cancer subtypes. **(E)** Differential copy number changes (Fisher's precision probability test pvalue<0.05) in 12 IWHMB-associated cancer subtypes. **(F)** TMB of 12 IWHMB-associated cancer subtypes. **(G)** CNV Burden of 12 IWHMB-associated cancer subtypes. **(H-J)** StromalScore, TumorPurity and ImmuneScore of 12 IWHMB-associated cancer subtypes. **(K)** Relationship between IWHMB-associated cancer subtypes and Kech subtypes. **(L)** DEGs of 12 IWHMB-associated cancer subtypes. **(M)** GSEA pathway enrichment of 12

IWHMB-associated cancer subtypes.

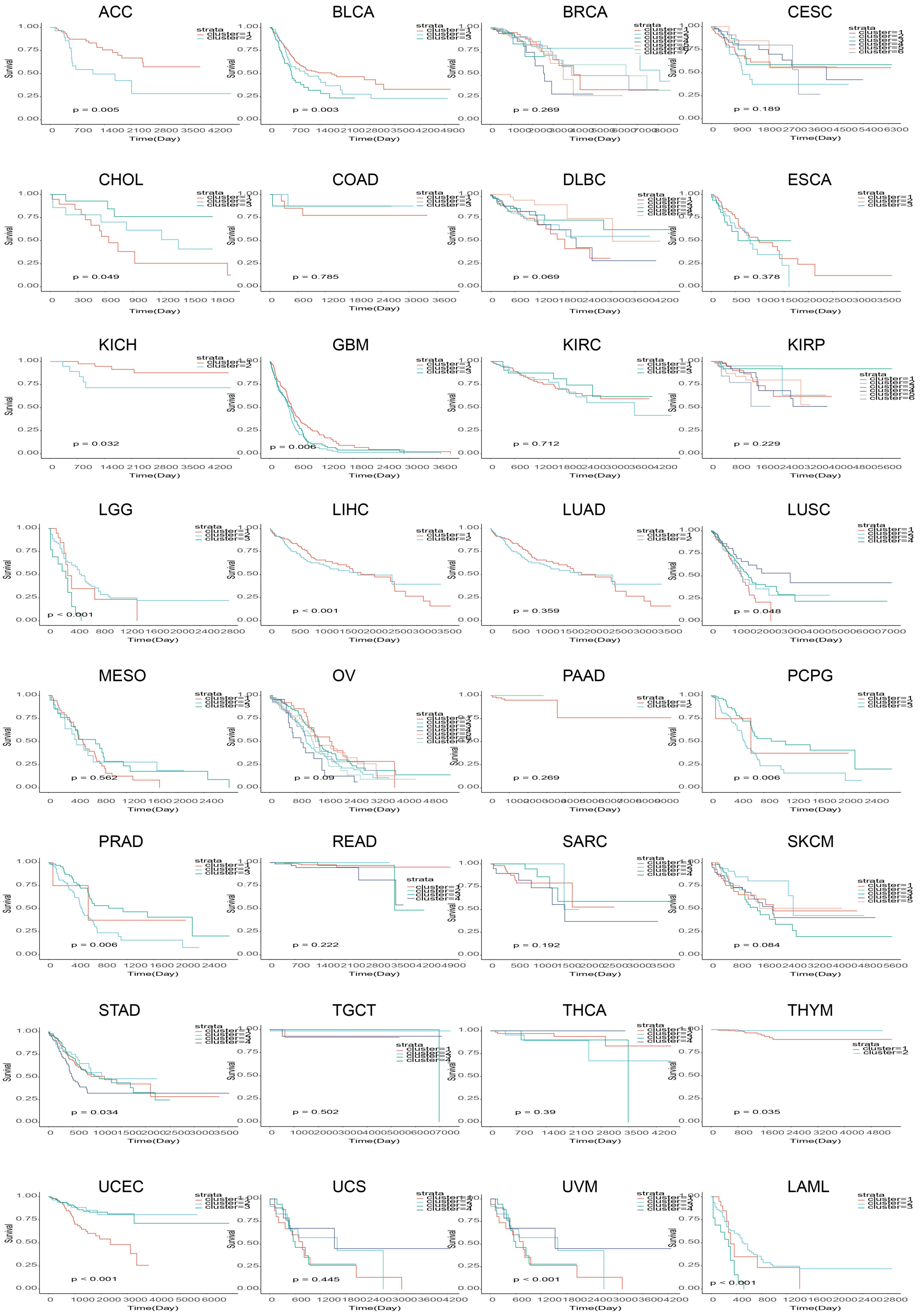

Fig. S4. Relationship between IWHMB-related subtypes and clinical prognosis in 32 TCGA cancers.

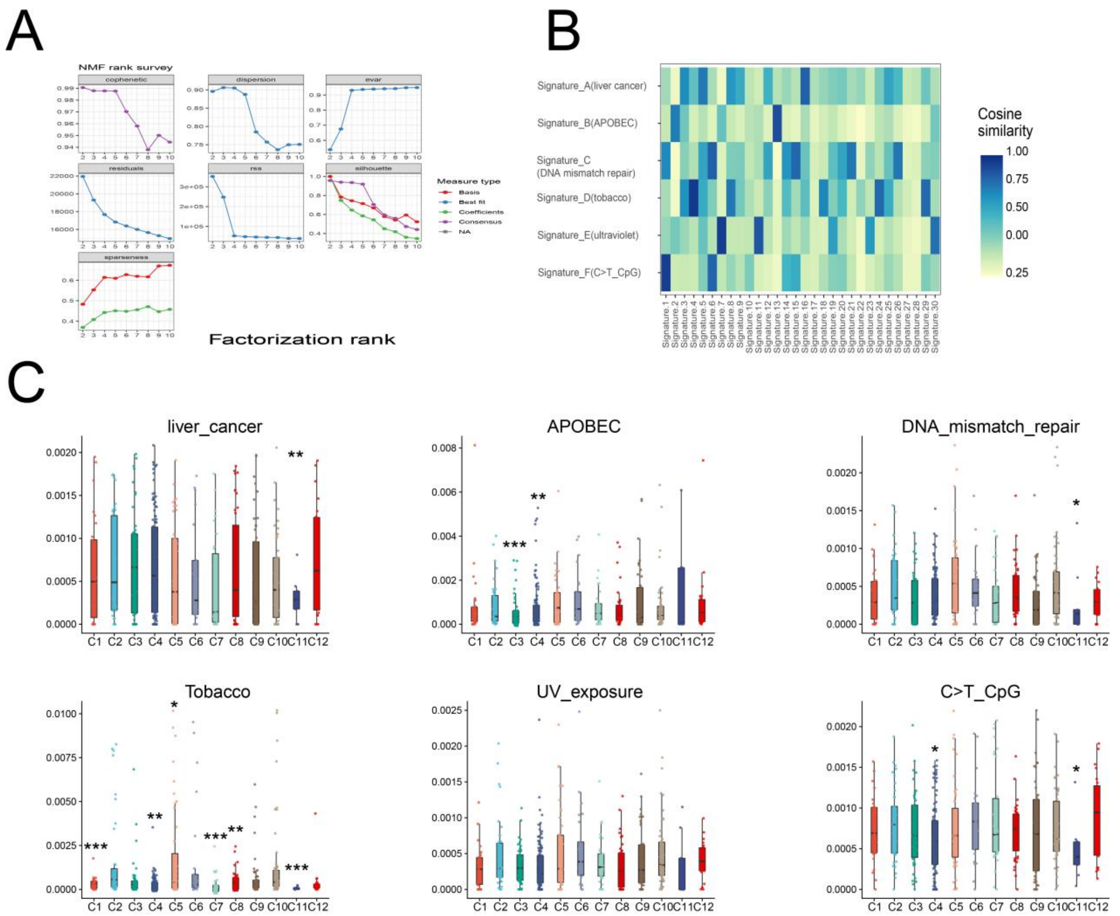

Fig. S5. Mutation Signatures in TCGA HNSCC cohort. **(A)** NMF of mutation signatures in the TCGA HNSCC cohort. **(B)** Cosine similarity of mutation signatures in the TCGA HNSCC cohort with annotated signatures recorded in the COSMIC database. **(C)** Association of 6 mutational signatures with IWHMB-related subtypes in the TCGA HNSCC cohort.

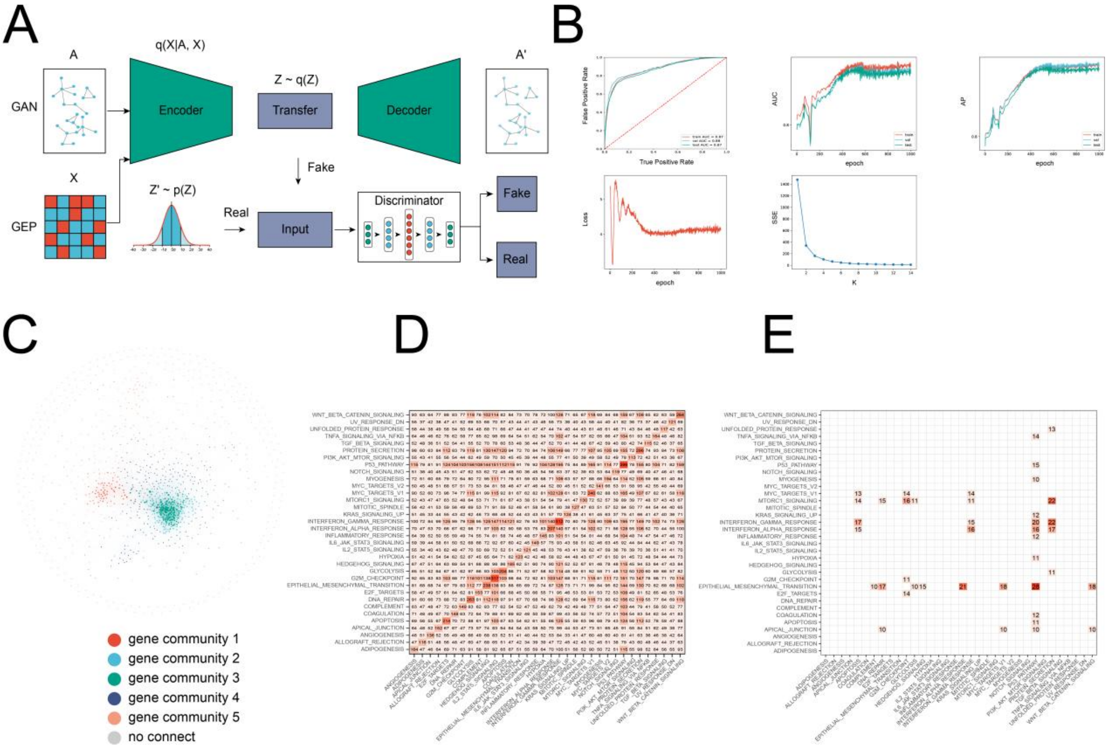

Fig. S6. ARGVA algorithm is used to identify Communities of IRG. **(A)** Schematic diagram of ARVGA algorithm. **(B)** Iteration period and loss function of ARVGA algorithm. **(C)** Communities visualization. **(D)** Interaction plot of 50 HGS disturbed genes. **(E)** Interaction plot of 50 HGS disturbed genes with the HGS itself.

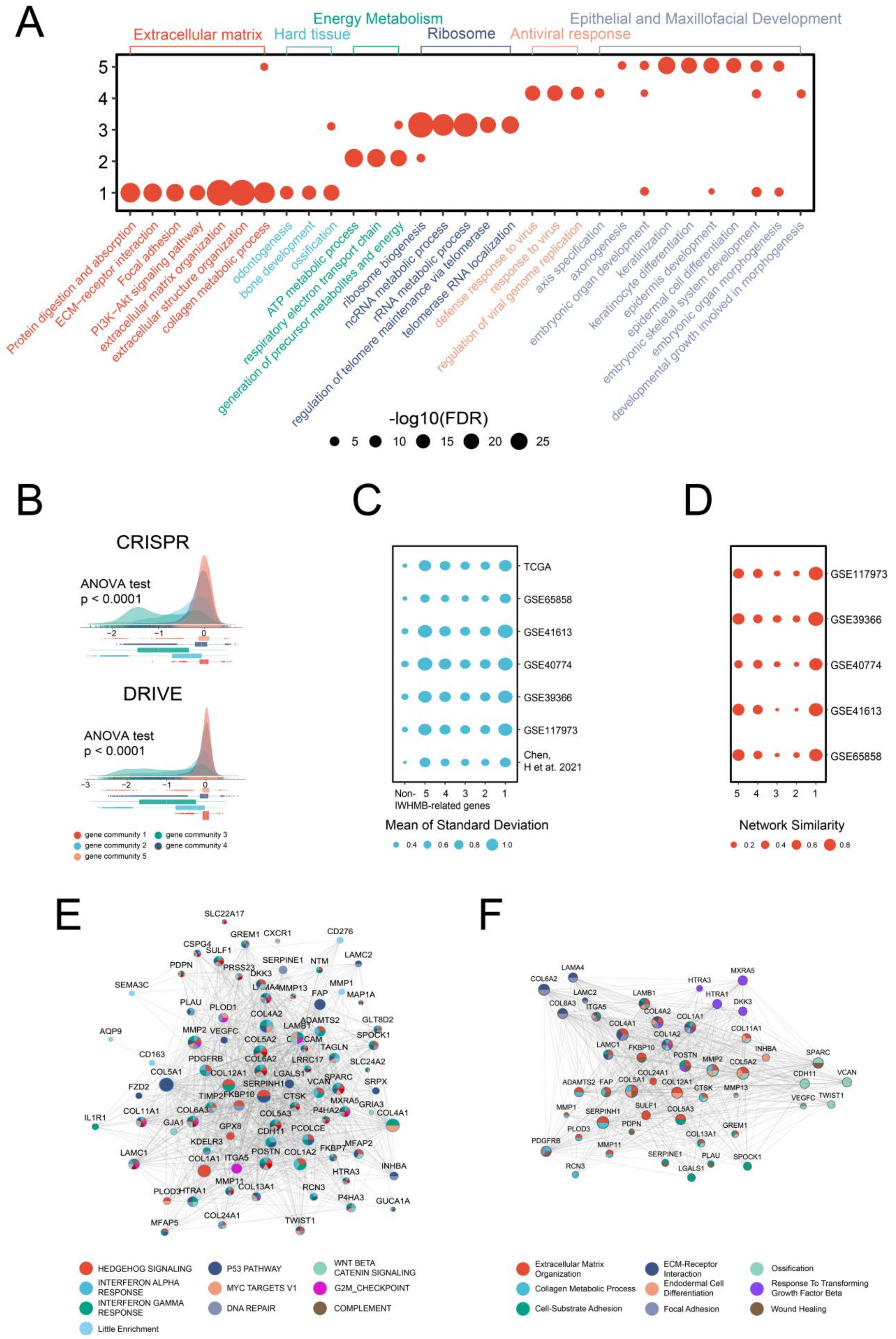

Fig. S7. Network properties of Communities. **(A)** GO and KEGG enrichment analysis of Communities. **(B)** The CRISPR-based or RNAi-based gene dependency scores between

Communities. **(C)** Average variability of Communities gene expression across multiple HNSCC cohorts. **(D)** Network similarity of Communities in multiple HNSCC cohorts. **(E)** Community 1 network visualization. Different colors represent that gene is interfered by different IWHMB of HGS. **(F)** Enriched path visualization in Community 1.

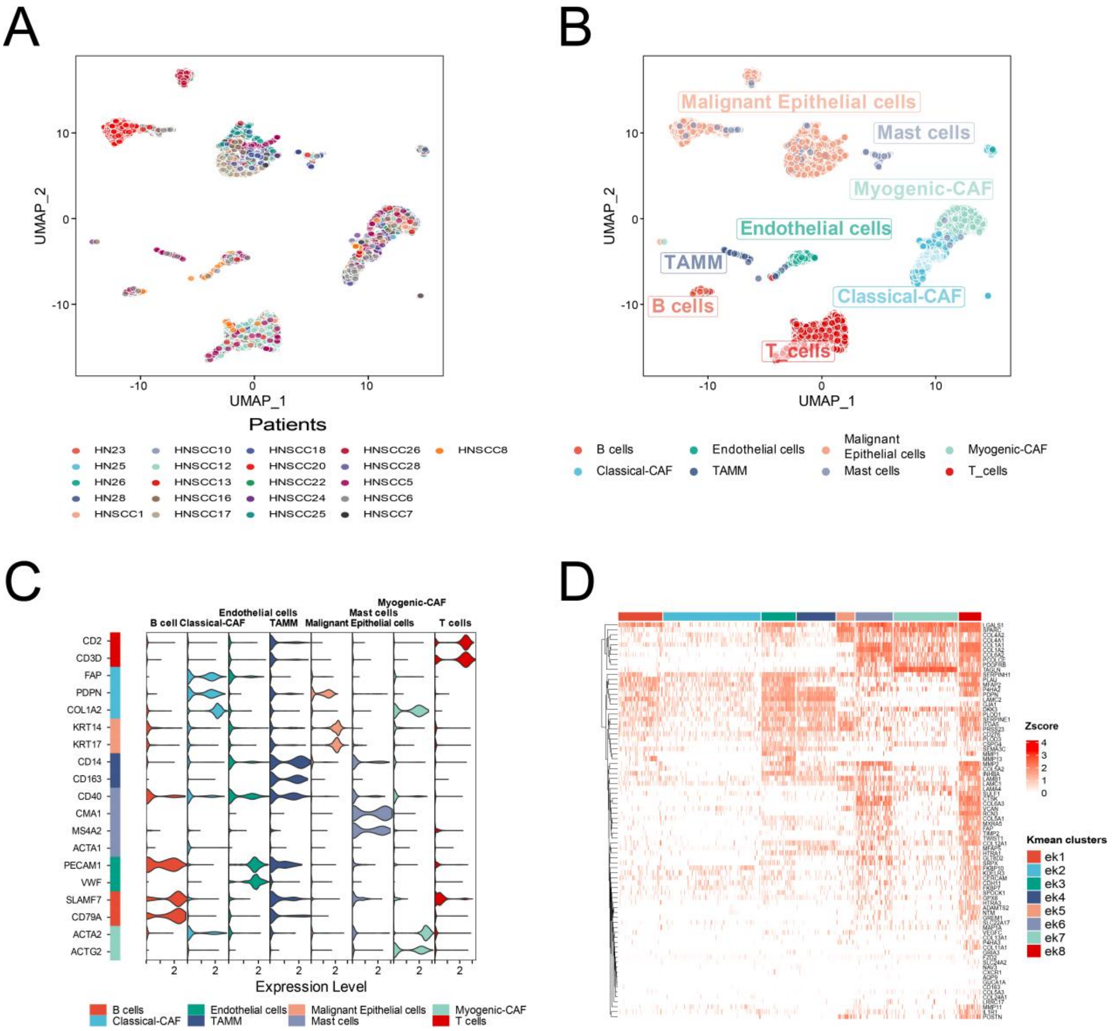

Fig. S8. Single cell annotation of GSE103322. **(A, B)** UMAP plot of GSE103322 single cells, colors represent tissue origin and cell type respectively. **(C)** Expression of marker genes in different cell types. (**D**) Kmean clustering of malignant epithelial, fibroblastic, and endothelial cells in GSE103322 using gene expression in community 1.

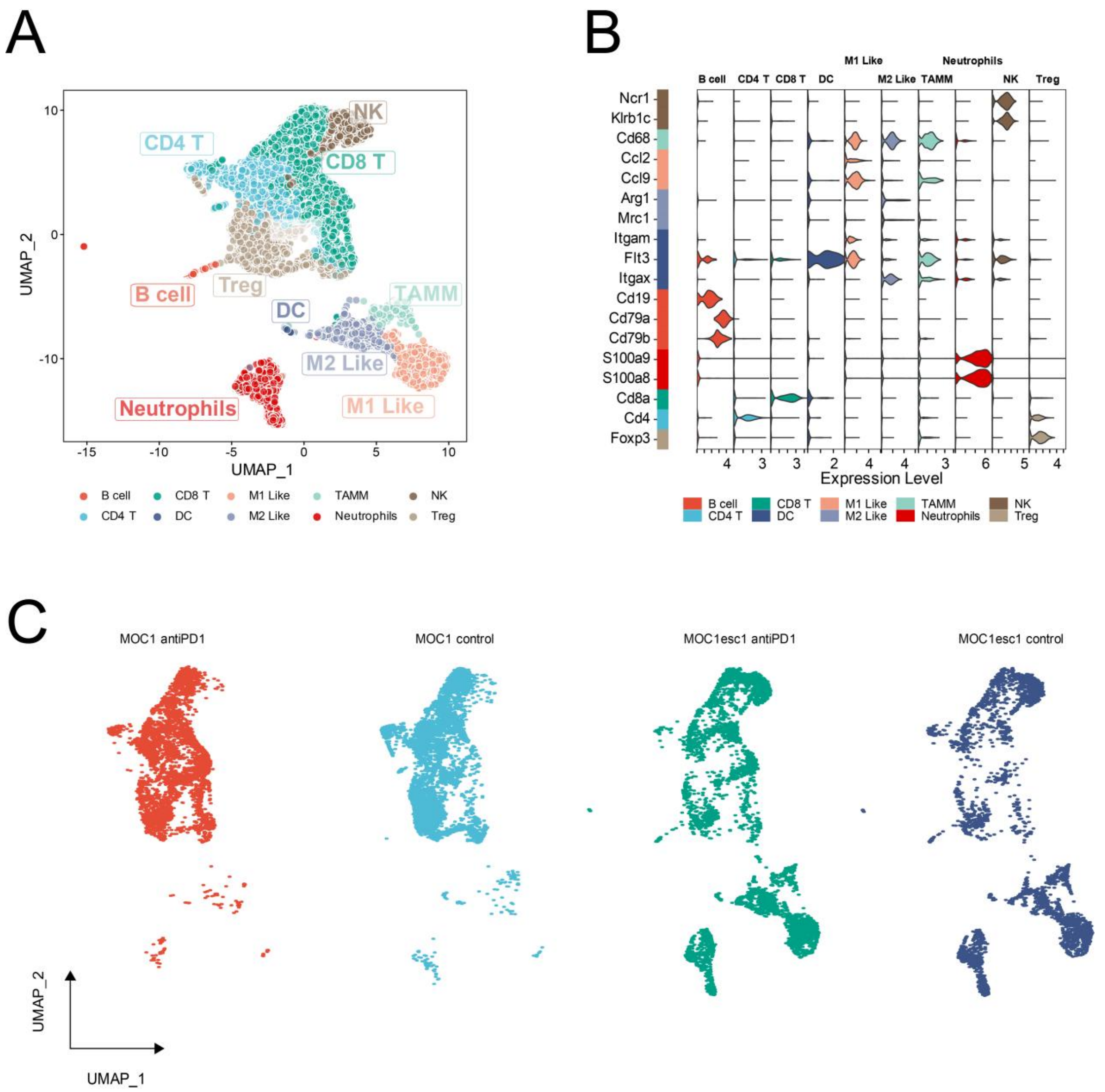

Fig. S9. Single cell annotation of GSE153383. **(A)** UMAP plot of GSE153383 single cells, colors represent cell type. **(B)** Expression of marker genes in different cell types. **(C)** Distribution of GSE153383 cells on UMAP plots under different conditions.

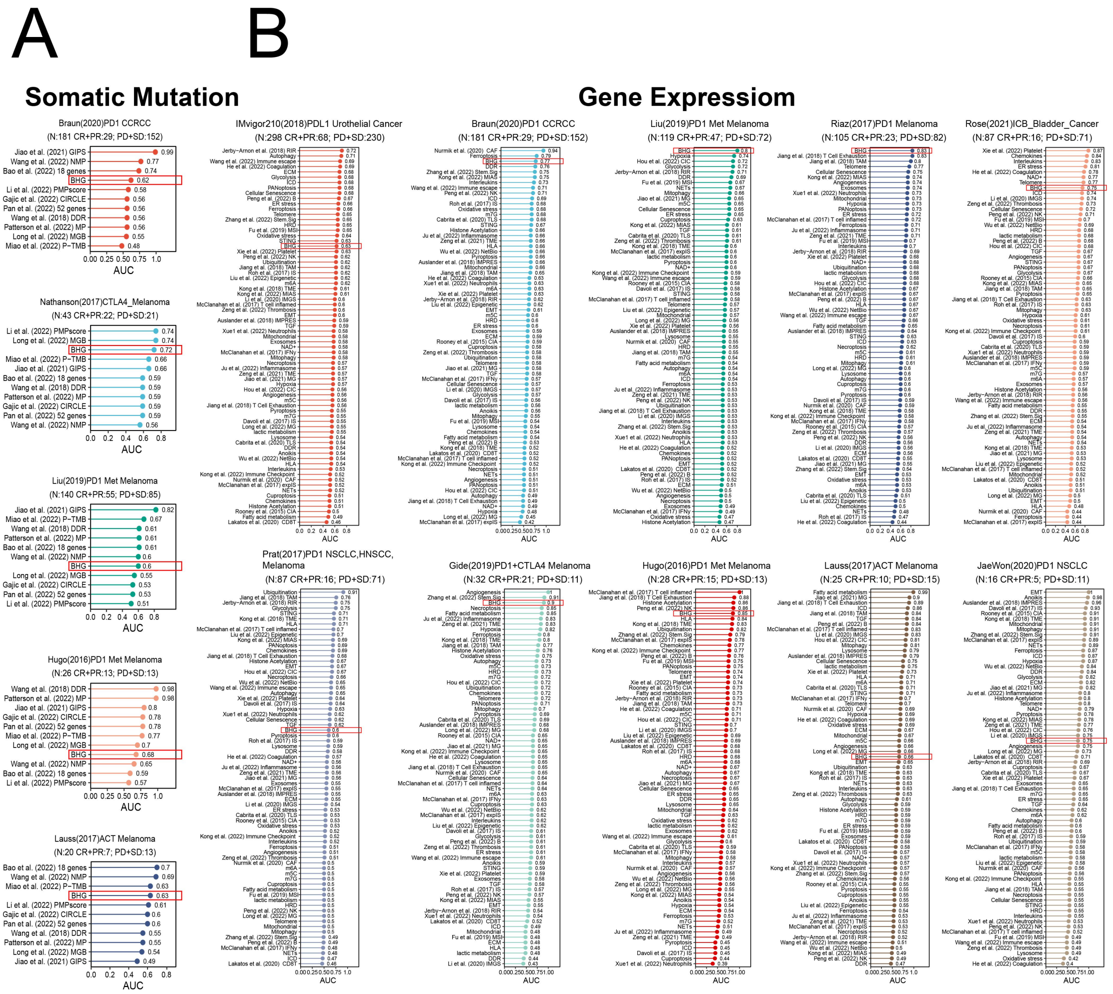

Fig. S10. Comparison of AUC values of BHG with other public gene signatures. **(A)** Comparison of AUC values of BHG with 10 gene signatures at the somatic mutation level for predicting ICI response in 5 ICI cohorts. **(B)** Comparison of AUC values of BHG with 70 gene signatures at the gene expression level for predicting ICI response in 10 ICI cohorts.
