# Supplementary material for: Quantified pathway mutations associate epithelial-mesenchymal transition and immune escape with poor prognosis and immunotherapy resistance of head and neck squamous cell carcinoma
Source: BMC Med Genomics. 2024 Feb 8;17:49. doi: 10.1186/s12920-024-01818-6 (PMC10854145; doi:10.1186/s12920-024-01818-6)
Supplement: Supplementary file 3 — Additional file 3: Supplementary methods. [file 12920_2024_1818_MOESM3_ESM.docx]

#### Supplementary Methods

**1. Identification of IWHMB-related genes**

To reduce interference, we retained the 34 HGSs in the TCGA cohort with WHMB in the sum of all samples >10, and then we performed a Support vector regression (SVR) with IWHMB of 34 HGSs as input and the zscore of gene expression (log2(TPM+1)) as response variable for each gene separately. The whole process was implemented using the function "svm" in R package e1071. Finally, the Spearman correlation between each gene predicted score and the zscore of gene expression (log2(TPM+1)) was used as the criterion for IWHMB-related genes (the process was firstly, the 21939 protein-coding genes annotated by ENSEMBLE in the TCGA cohort were modeled and retained according to r>0.2, and then validated these models with the Chen’s cohort and retained according to r>0.1). Finally, 1031 IWHMB-related genes were identified, and in addition we generated a null distribution of parameters to verify that the achieved performance was "real" and not by random chance, specifically, gene expression values are randomly from gene expression matrix to create random matrix, by which SVR regression learning is performed for 1000 iterations to obtain the null distribution of parameters distribution.

**2. Network Communities detection**

We obtained Gene and Protein interaction network (TF-Target & PPI, the details are described in the results) of 1031 IWHMB-related genes. We adopted the Adversarially Regularized Graph Autoencoder (ARVGA) proposed by Pan et al. (2019) ^1^ for Network Communities detection, which is a deep learning method combining Graph Variational Auto-Encoders and Adversarial neural Network. Specifically, the algorithm is divided into twox` parts:

Encoder:

$$\begin{aligned} Z_{l+1}=f\left( Z_{l}, A | W_{l} \right) \# \left( 1 \right) \end{aligned}$$

$$\begin{aligned} f\left( Z_{l}, A | W_{l} \right)=\emptyset\left( \tilde{D}^{-\frac{1}{2}}\tilde{A}\tilde{D}^{-\frac{1}{2}}Z_{l}W_{l} \right) \# \left( 2 \right) \end{aligned}$$

$$\begin{aligned} q\left( Z | X, A \right)=\prod_{i=1}^{n} q\left( z_{i} | X, A \right) \# \left( 3 \right) \end{aligned}$$

where formula 1 represents the most basic layer-wise transformation process of graph neural network, Z initial value Z0 represents the node attribute feature (Gene Expression Matrix, Z), A is the Gene and Protein interaction network, $\tilde{A}=A+I$, $I$ is the identity matrix of A, $\tilde{D}=\sum_{j} \tilde{A}_{jj}$, $\emptyset$ is an activation function (Reln is used in the article), W is the matrix of parameters to be learned, $q\left( z_{i} | X, A \right)=N\left( z_{i} | \mu_{i}, diag(\sigma^{2}) \right)$. The encoder in this article adopts a three-layer structure, and the number of neurons in each layer is 32, 64, 32.

Decoder:

$$\begin{aligned} p\left( \hat{A} | X \right)=\prod_{i}^{n} \prod_{j}^{n} p\left( \hat{A}_{ij} | z_{i},z_{j} \right)\#\left( 4 \right) \end{aligned}$$

$p\left( \hat{A}_{ij} | z_{i},z_{j} \right)=sigmoid({z_{i}}^{T},z_{j})$

where we use predicted A (A ̂) to reconstruct graph structure A, $p\left( \hat{A}_{ij} | z_{i},z_{j} \right)$ by predicting the probability whether there is a connection between node i and node j

Adversarial Mode: A standard three-layer feedforward neural network with 32, 64, 32 neurons in each layer and a sigmoid function for the last layer.

This algorithm optimizes a total of two functions, 1 The loss function of variational graph encoder is defined as:

$$\begin{aligned} L1=E_{q\left( Z | \left( X, A \right) \right)}\left[ \log p\left( \hat{A} | X \right) \right]-KL[q\left( Z | X, A \right)||(p\left( Z \right) \#(5) \end{aligned}$$

where KL is Kullback-Leibler divergence between two distributions, $p\left( Z \right)=\prod_{i=1}^{n} q\left( z_{i} | 0, I \right)$. 2 The loss function of Adversarial Mode is defined as:

$$\begin{aligned} L2=-\frac{1}{2}E_{z\sim p_{z}}logD\left( Z \right)-\frac{1}{2}E_{X}\log\left( 1-D\left( G\left( X, A \right) \right) \right) \#(6) \end{aligned}$$

where D is Adversarial Mode, $G(X, A)$ is negative sample transformed by Encoder.

Communities are obtained by k-mean clustering of the encoded output matrix.

**3. Network Node prioritization.**

Random walk with restart algorithm (RWR) ^2^ were used for node priorization. The approach of RWR is as follows: the seed genes are set, and then over iterate by randomly walking to neighbor nodes with restart probability γ. The iteration formula is:

$$\begin{aligned} P_{t+1}=\left( 1-\gamma\right)P_{t}+\gamma P_{0}\#\left( 7 \right) \end{aligned}$$

where $P_{0}$ is the weight of all initial nodes, set to 1 in the article, W is the column-normalized adjacency matrix of all nodes in the network, and $\gamma$ is the restart probability, set to 0.75 in the article. After a certain number of iterations, Pt reaches a particular probability distribution, $P_{t+1}$ is almost equal to $P_{t}$. $P_{t}$ represents the network proximity score of the other nodes in the network and the seed nodes.

**4. HNSCC scRNA-seq Data Analysis**

**GSE103322**

**4.1. Quality control, Dimensionality reduction and clustering**

We retained genes expressed in at least 3 cells and cells with at least 200 genes expressed, a total of 5676 cells, and 21519 genes were retained. We then generated a high-variance matrix (containing only the top 3000 genes with the highest expression changes), performed principal component analysis (PCA) to reduce the dimensionality of high-variance matrix, extracted the top 30 principal components for The Uniform Manifold Approximation and Projection (UMAP) analysis and Clustering analysis for all cells.

**4.2. Cell annotation**

We used CellMarker database and the marker provided in the literature ^3^ for cell annotation of different Clusters

**4.3. Community1-related cell Clustering**

We used the 80 Community1 genes obtained above for K-means clustering for cells annotated to epithelial cells and stromal cells, centers are set to 8.

**4.4. Pseudotime analysis**

We performed trajectory analysis of the annotated malignant epithelial cells and stromal cells (endothelial cells and cancer associated fibroblasts) using monocle2 (here we selected 3000 genes that are highly variable in the above cells for pseudotime ordering).

**4.5. Cellular communication**

We used CellPhoneDB (Python version) to estimate the significance of ligand-receptor pairs in different cell types, retaining ligand-receptor pairs with pvalue < 0.05.

**4.6. AddModuleScore**

We used the Seurat package function AddModuleScore to Calculate the average expression levels of gene set on single cell level.

**4.7. Gene Regulatory Network of transcription factors (TFs) Analysis**

We used pySCENIC (SCENIC python version) to construct TF-Target network and infer TF-AUC values of TFs in each cell, this analysis is based on all cells, hg19 is based on all cells, hg19-tss-centered-10kb-10species.mc9nr.feather as gene-motif rankings

**GSE153383**

**4.8. Quality control and integration**

We retained genes expressed in at least 3 cells and cells with at least 200 genes expressed, a total of 16885 cells, and 17366 genes were retained. By screening out 3000 genes that were highly variable in each of MOC1 and MOC1, two functions "FindIntegrationAnchors", "IntegrateData" in R package Seurat were used to integrate the two cell line datasets.

**4.9. Dimensionality reduction and clustering**

We extracted the top 30 principal components for The Uniform Manifold Approximation and Projection (UMAP) and Cell clustering

**4.10. Cell annotation**

We took two approaches to annotation, 1 CellMarker database and literature ^4^ were used to provide Marker genes for cell annotation. 2 For cells annotated as T cells (CD8 T cells, CD4 T cells and Treg), ProjecTILs algorithm were used to re-annotation.

**4.11. Identification of Community1-related Immune genes**

We treated immune genes from ImmPort and InnateDB database which absolute correlation coefficient with MEC1>0.3 and pvalue<0.05 in at least two HNSCC cohorts as Community1-related Immune genes, and obtained a total of 497 genes. Then homologous conversion of mouse and human genes to SYMBOL id was performed using R package homologene.

**4.12. CD8 T cells re-clustering based on Community1-related Immune genes**

We obtained the intersection of Community1-related Immune genes and 1000 CD8 T cells high variant genes and extracted the top 30 genes, and then performed K-means clustering based on these 30 genes. cell clustering, centers are set to 8.

**4.13. Diffusion map analysis**

Single cells assigned to CD8 T cells were used to Diffusion map, 3000 CD8 T cells high variant genes were used to recalculate principal The whole process was implemented using the R package Destiny, "DiffusionMap", with parameters set to n_eigs = 20, n_pcs = 50, and the rest of the parameters as default values

**4.14. Cellular communication**

We used CellPhoneDB (Python version) to estimate the significance of ligand-receptor pairs in different cell types, keeping pvalue < 0.05 and containing Community1- related Immune genes of ligand-receptor pairs.

**5. HNSCC TCR-seq Data Analysis**

Processing of 10X immunoassays with the R package Immunarch Pipeline. Specifically, the number of clonetypes is detected using the "repExplore" function, and Diversity estimation is performed using "repDiversity", methods=hill..

**6. Classified Machine Learning Model for Predicting ICI Responses**

In the Braun et al. (2020 antiPD1 CCRCC) cohort, 5 machine learning models (RF, SVM, lasso, Ridge and Xgboost) were selected to compare the predictive ability of BHG for ICI responses at three Omics levels. Cross-validation was Leave-One-Out (LOO), Principal Component Analysis (PCA) was used to reduce the effect of multicollinearity, and Manual Variable Filtering (MVF) at each training session was used to increase the robustness of the models. The approach of MVF is as follows: we divide the cohort into response (CR and PR) and resistance (PD and SD) and set a threshold (0.05 by default). If the P value of the difference test of variable between the two groups is less than the threshold, the variable is regarded as a hyper-variable. If the number of hyper-variables is less than 2, the threshold is set to 1, which mean that all variables are retained for training. If the number of hyper-variables is greater than 8, all hyper-variables are retained for training. If the number of hyper-variables is between 2 and 8, threshold is set to 0.3 and all variables that meet the conditions are retained. 11 ICI cohorts were used to compare the predictive power of BHG with other gene signatures. Centralization and PCA are used for data processing. However, we found that the AUC of BHG was 0.46 and 0.52 for Liu et al. (2019 anti PD1 Met Melanoma) and JaeWon et al. (2020 anti PD1 NSCLC) cohorts, respectively. The results suggest that BHG fail to learn the internal biological features of the cohorts. We speculate that centralization removes some of the biological features from the data, so we did not centralize the data in the two cohorts but kept the original features of the data. The model was performed by lasso regression, cross-validation by LOO, and MVF as before.

1. Pan, S.; Hu, R.; Fung, S. F.; Long, G.; Jiang, J.; Zhang, C., Learning Graph Embedding With Adversarial Training Methods. *IEEE Trans Cybern* **2020,** *50* (6), 2475-2487.

2. Valdeolivas, A.; Tichit, L.; Navarro, C.; Perrin, S.; Odelin, G.; Levy, N.; Cau, P.; Remy, E.; Baudot, A., Random walk with restart on multiplex and heterogeneous biological networks. *Bioinformatics* **2019,** *35* (3), 497-505.

3. Puram, S. V.; Tirosh, I.; Parikh, A. S.; Patel, A. P.; Yizhak, K.; Gillespie, S.; Rodman, C.; Luo, C. L.; Mroz, E. A.; Emerick, K. S.; Deschler, D. G.; Varvares, M. A.; Mylvaganam, R.; Rozenblatt-Rosen, O.; Rocco, J. W.; Faquin, W. C.; Lin, D. T.; Regev, A.; Bernstein, B. E., Single-Cell Transcriptomic Analysis of Primary and Metastatic Tumor Ecosystems in Head and Neck Cancer. *Cell* **2017,** *171* (7), 1611-1624 e24.

4. Zhou, L.; Zeng, Z.; Egloff, A. M.; Zhang, F.; Guo, F.; Campbell, K. M.; Du, P.; Fu, J.; Zolkind, P.; Ma, X.; Zhang, Z.; Zhang, Y.; Wang, X.; Gu, S.; Riley, R.; Nakahori, Y.; Keegan, J.; Haddad, R.; Schoenfeld, J. D.; Griffith, O.; Manguso, R. T.; Lederer, J. A.; Liu, X. S.; Uppaluri, R., Checkpoint blockade-induced CD8+ T cell differentiation in head and neck cancer responders. *J Immunother Cancer* **2022,** *10* (1).
